# Proteogenomic characterization and mapping of nucleosomes decoded by Brd and HP1 proteins

**DOI:** 10.1186/gb-2012-13-8-r68

**Published:** 2012-08-16

**Authors:** Gary LeRoy, Iouri Chepelev, Peter A DiMaggio, Mario A Blanco, Barry M Zee, Keji Zhao, Benjamin A Garcia

**Affiliations:** 1 Department of Molecular Biology, Princeton University, 415 Schultz Laboratory, Princeton NJ 08544, USA; 2 Systems Biology Center, National Heart, Lung and Blood Institute, National Institutes of Health, Bethesda, MD 20892, USA; 3 Epigenetics Program, Department of Biochemistry and Biophysics, Perelman School of Medicine, University of Pennsylvania, 1009C Stellar-Chance Laboratories, 422 Curie Boulevard, Philadelphia, PA19104, USA; 4 Department of Chemistry, Princeton University, Princeton NJ 08544, USA; 5 Quantitative and Computational Biology Program, Princeton University, Princeton, NJ 08544, USA

## Abstract

**Background:**

Histone post-translational modifications (PTMs) constitute a branch of epigenetic mechanisms that can control the expression of eukaryotic genes in a heritable manner. Recent studies have identified several PTM-binding proteins containing diverse specialized domains whose recognition of specific PTM sites leads to gene activation or repression. Here, we present a high-throughput proteogenomic platform designed to characterize the nucleosomal make-up of chromatin enriched with a set of histone PTM binding proteins known as histone PTM readers. We support our findings with gene expression data correlating to PTM distribution.

**Results:**

We isolated human mononucleosomes bound by the bromodomain-containing proteins Brd2, Brd3 and Brd4, and by the chromodomain-containing heterochromatin proteins HP1β and HP1α. Histone PTMs were quantified by mass spectrometry (ChIP-qMS), and their associated DNAs were mapped using deep sequencing. Our results reveal that Brd- and HP1-bound nucleosomes are enriched in histone PTMs consistent with actively transcribed euchromatin and silent heterochromatin, respectively. Data collected using RNA-Seq show that Brd-bound sites correlate with highly expressed genes. In particular, Brd3 and Brd4 are most enriched on nucleosomes located within HOX gene clusters, whose expression is reduced upon Brd4 depletion by short hairpin RNA.

**Conclusions:**

Proteogenomic mapping of histone PTM readers, alongside the characterization of their local chromatin environments and transcriptional information, should prove useful for determining how histone PTMs are bound by these readers and how they contribute to distinct transcriptional states.

## Background

A cell's transcriptional program is governed not only by *cis*-acting DNA sequences, but also by chromatin structure, DNA methylation and histone post-translational modifications (PTMs). Chromatin modifications and the gene expression patterns that accompany them are maintained when a cell divides and are thus known as 'epigenetic' [1,2]. Histone lysine methylation is associated with both transcribed and non-transcribed chromatin. For example, H3K4me3 is found in the 5' region of active genes, whereas H3K36me2 and H3K36me3 are enriched within the body and the 3' end of active genes, respectively [3,4]. However, H3K9 and H3K27 trimethylation are implicated in both the formation and spreading of constitutive heterochromatin and silencing of euchromatin. In contrast to methylation, histone H3 and H4 acetylation is a general characteristic of transcriptionally active chromatin. Depending on the residue modified, acetylations can be localized to nucleosomes near the 5' promoter region or enriched throughout coding regions of actively transcribed genes. For instance, chromatin immunoprecipitation experiments have shown that H3K27ac and H3K14ac are enriched on only a few nucleosomes at the promoters of active genes, whereas H4 acetylations correlate with large euchromatic regions containing transcribed loci [3,5].

The 'histone code' hypothesis proposes that specific histone PTMs encode regulatory information that is read by the binding of accessory proteins. These accessory proteins, termed 'readers', bind via specialized histone-PTM-binding domains such as bromodomains, chromodomains and plant homeodomains (PHDs) [1]. The bromodomain binds directly to acetylated lysines in histones and is commonly found in proteins associated with gene activation, such as Brg1, a subunit of the hSWI/SNF remodeling complex, the acetyltransferase hGcn5, and the Brd proteins, which are mammalian homologs of the *Drosophila *trithorax Fsh1 and Fsh2 proteins [6]. The Brd2, Brd3 and Brd4 proteins are referred to as BET proteins as they contain tandem bromodomains and an extraterminal domain of unknown function [7]. *In vitro *studies have demonstrated that Brd2 and Brd3 possess nucleosome chaperone activities that allow RNA polymerase II to elongate transcripts through hyperacetylated nucleosomes, implicating their direct role in transcription [8]. Several reports have also implicated Brd regulation of cell cycle progression and inflammatory response [9,10]. Thus, the unique protein architecture and function of Brd proteins qualify them as promising targets for selective pharmacological inhibitors such as immunosuppressants and anticancer drugs. In fact, recent publications have reported two Brd protein selective small molecule inhibitors (I-BET and JQ-1) that specifically inhibit bromodomain binding to acetylated histones, thereby blocking a lipopolysaccharide-induced cytokine storm and the growth of Brd-dependent tumors [9,11].

In contrast to bromodomains, chromodomains are methyl-lysine binding domains found in proteins such as the heterochromatic proteins HP1α and HP1β that bind to H3K9me3 [12]. Chromodomains are also found in the polycomb group, CBX proteins, which mediate silencing by packaging specifically methylated nucleosomes into heterochromatin-like clusters [13]. In addition to a chromodomain, HP1α and HP1β contain a chromoshadow domain thought to be involved in interactions with other proteins [14]. The HP1α and HP1β proteins form both homo- and heterodimers, and although these proteins colocalize at many heterochromatic loci, they also bind to distinct loci [15].

**Figure 1:**
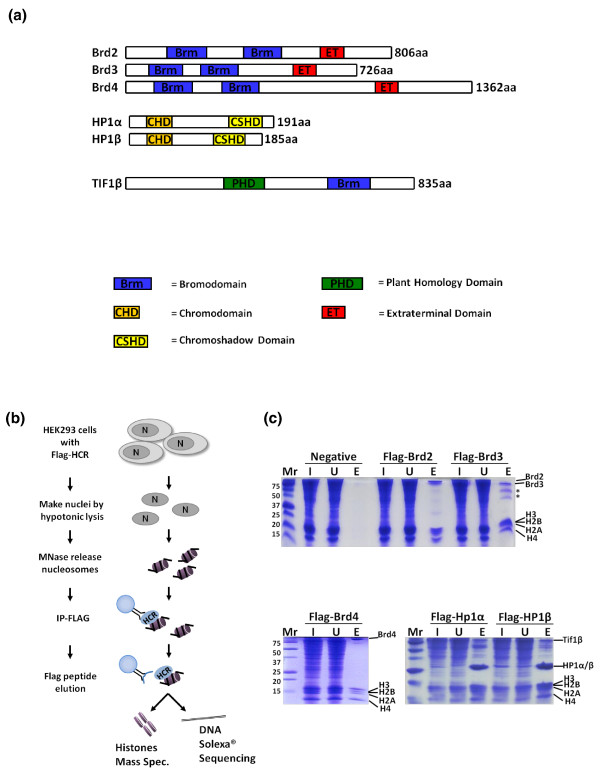
**Isolation of Brd- and HP1-bound mononucleosomes**. **(a) **Illustration of the domain architecture of the histone code readers. **(b) **Schematic of protocol used to isolate nucleosomes bound to FLAG-histone code readers (HCRs). **(c) **Visualization of FLAG-Brd- and FLAG-HP1-bound nucleosome purifications. The proteins present in the input (I), the immunoprecipitation unbound (U), and FLAG-peptide-eluted (E), fractions were examined by SDS-PAGE and Coomassie blue staining. The asterisk denotes Brd3 breakdown products detected by liquid chromatography-tandem mass spectrometry.

Here, we report experiments that elucidate the combinations of histone PTMs on nucleosomes associated with the histone code reading proteins Brd2, Brd3, Brd4, HP1α and HP1β (Figure 1a), applying a chromatin immunoprecipitation quantitative mass spectrometry (ChIP-qMS) approach. We also utilized deep sequencing to map the DNA sequences contained within these nucleosomes to their genomic locations and used RNA sequencing and microarrays to determine the transcriptional state of the nucleosomes. Knockdown of Brd4 or HP1β suggests that the proper expression of many genes is dependent on their associated Brd or HP1 proteins. Providing genomic maps of where histone code readers are bound and the modifications found on such nucleosomes lays down the foundation that will aid future work aimed at deciphering how the network of chromatin-associated proteins 'translate' these histone PTMs. Additionally, these proteomic ChIP-qMS methods can be generally applied to any chromatin binding protein to characterize their local chromatin environments and identify enriched histone PTM patterns. Combined with further downstream analyses we are able to correlate this information to transcriptional states.

## Results

**Figure 2:**
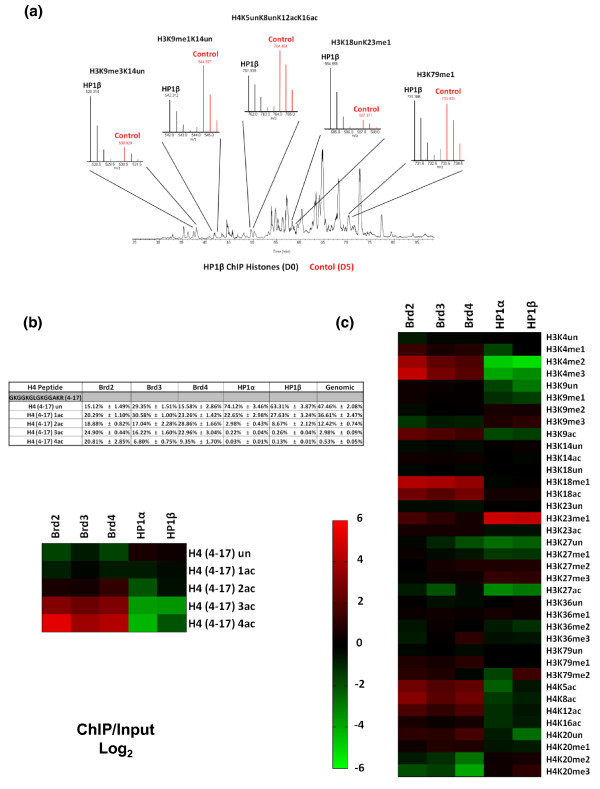
**Histone H3 and H4 residue-specific PTMs quantified by mass spectrometry**. **(a) **Total ion chromatogram and full MS spectrum of propionylated histone peptides used for quantification from an HP1β ChIP; ChIP elution D0 labeled (black), and the control ChIP input D5 labeled (red). Full MS spectrum of peptides shown; H3K9me3K14un, H3K9me1K14un, H4K5unK8unK12acK16ac, H3K18unK23me1 and H3K79me3. **(b) **Quantification of the degree of acetylation of the histone H4 peptide (amino acids 4 to 17, GKGGKGLGKGGAKR). Values in the table are the total percentage of each acetylated form and the heatmap is a representation of the fold change of the specified ChIP/Input in (log_2_) scale. **(c) **Heatmap depicting all modifications quantified on the histone H3 and H4 proteins. Values used to generate the heatmap are found in Additional file 2. The heatmap was generated with the fold change values of each PTM for each specified ChIP/Input in (log_2_) scale.

**Figure 3:**
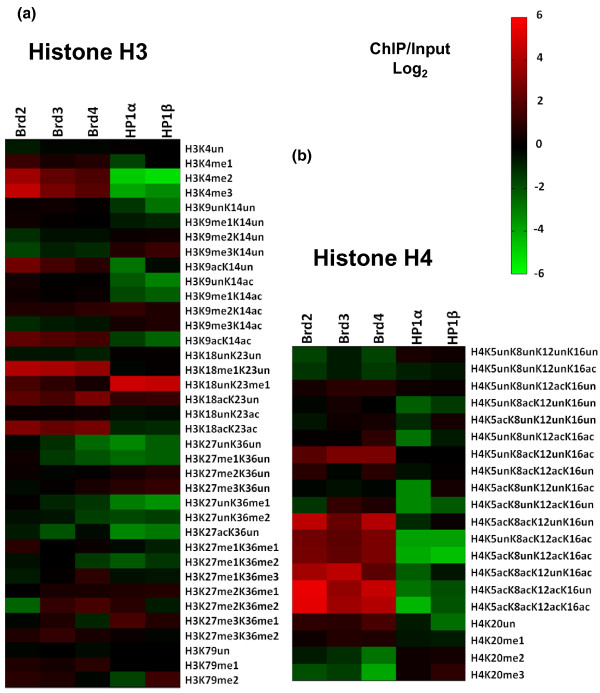
**Quantification of PTMs on histone H3 and H4 peptides isolated with HP1 and Brd proteins**. **(a) **Heatmap depicting all modified forms of H3 peptides on HP1- and Brd-bound nucleosomes by qMS. Values used to generate the heatmap are in Additional files 2 and 6. The heatmap was generated with the fold change values for each modified H3 peptide quantified from each specified ChIP/Input in (log_2_) scale. **(b) **Heatmap depicting all modified forms of H4 peptides on HP1- and Brd-bound nucleosomes by mass spectrometry. Values used to generate the heatmap are in Additional files 2 and 3. The heatmap was generated with the fold change values for each modified H4 peptide quantified from each specified ChIP/Input in (log_2_) scale.

To gain insight into the local chromatin make-up of histone PTM binding proteins, we optimized a ChIP technique to purify mononucleosomes from cells expressing tagged chromodomain- and bromodomain-containing proteins. Specifically, we generated HEK293 cell lines that express FLAG-tagged fusions of Brd2, 3, 4 and HP1α and β (Figure 1a), as these bromodomain- and chromodomain-containing proteins are believed to mitigate opposing epigenetic functions and could provide solid proof of principle experiments. Western blots (Additional file 1) show that our transiently expressed proteins are above endogenous. As outlined in Figure 1b, following nuclear isolation and micrococcal nuclease digestion, nucleosomes bound by the various FLAG-tagged proteins were immunoprecipitated and competitively eluted with excess FLAG peptide. Figure 1c shows that FLAG-tagged Brds and HP1s all immunoprecipitate the core histones while no histones were detected from cells not expressing a FLAG-tagged protein. We find that competitive elution with FLAG peptide reduces non-specific binding compared to boiling the beads in SDS buffer. Immunoprecipitates from FLAG-tagged HP1α and HP1β contained other abundant polypeptides, such as Tif1β protein, a heterochromatic protein that is known to interact and colocalize with HP1α and β [16,17]. Interestingly, Tif1β contains a bromodomain and PHD domain (Figure 1a), which may allow for a more complex histone code to be read when in complex on a nucleosome with HP1. The immunoprecipitated histones were then derivatized with propionic anhydride, trypsinized, and subjected to qMS via nano scale liquid chromatography-tandem mass spectrometry (nanoLC-MS/MS) experiments on an Orbitrap instrument [18]. The derivatization allows for the incorporation of isotopically stable *d0*- or *d5*-propionyl groups such that two samples (each labeled with a different isotope) can be analyzed together in a single nanoLC-MS/MS experiment (Figure 2a) or samples can be both labeled with *d0*-propionyl and run individually. The histone modifications found on H3 and H4 peptides from a ChIP-qMS and whole-genome control are then quantified using in-house developed software (see Materials and methods) as depicted in Figures 2 and 3.

### Characterization of PTMs on histone H4 from Brd- and HP1-associated nucleosomes

PTMs observed on the H4 molecules from Brd-bound nucleosomes contained marks consistent with gene activation. Most striking was the enrichment in the overall degree of acetylation. All the potentially acetylated lysines (K5, K8, K12 and K16) occur on the same propionylated tryptic peptide, so we first quantified H4 tail peptides that were unmodified, mono, di-, tri- and tetra-acetylated, irrespective of the residue modified (Figure 2b, table). When compared to whole genome chromatin, all Brd nucleosomes were enriched in H4 acetyl modifications; 1.3- to 2.3-fold for the diacetylated species, 5.4- to 8.3-fold for the triacetylated species and 12.9- to 39.5-fold for the tetracetylated form (Figure 2b, heatmap). Notably, the Brd nucleosomes contained only 15 to 29% unacetylated H4 molecules as opposed to genomic histone H4, which was 50% unacetylated. As nucleosomes contain two copies of each histone, the unacetylated species within these nucleosomes is probably not directly bound by the Brds. On the other hand, HP1α- and HP1β-bound nucleosomes had relatively low levels of H4 acetyls. In fact, approximately 70% of the H4 tails were unmodified on HP1-bound nucleosomes (Figure 2b, table). However, the HP1 bound nucleosomes have moderate levels of H4 acetylation, such as tails with a monoacetyl (approximately 25%). The single most abundant H4 tail acetylation in HEK 293 cells is H4K16, occurring in approximately 41% of all histone H4 molecules (Additional file 2). This particular acetylation mark was only slightly enriched on Brd nucleosomes (1.2- to 1.5-fold) (Figure 2c). We suspect that Brd proteins may not directly read this PTM; rather, its slight enrichment in Brd nucleosomes is a consequence of being associated with other active chromatin marks. Nucleosomes bound by all three Brd proteins were significantly enriched in acetylations at the other sites on histone H4; H4K5ac (4.7- to 6.3-fold), H4K8ac (4.1- to 8.7-fold) and H4K12ac (2.3- to 3.3-fold) (Figure 2c) consistent with X-ray crystallography structural studies [19,20].

Our in-house software (see Materials and methods) accurately deconvoluted the mixed tandem mass spectra resulting from isobaric acetylation combinations that occur on H4 (that is, K5, K8, K12 and K16). When compared to whole genome chromatin, nucleosomes bound by all three Brd proteins were enriched with the H4 diacetylated forms (K5unK8acK12unK16ac, K5unK8acK12acK16un and K5acK8unK12unK16ac), while only Brd3- and Brd4-bound nucleosomes were enriched in the K5acK8unK12acK16un species (Figure 3b, heatmap; Additional file 3). These results suggest that Brds will tolerate H4 containing K16ac, although it is not required for binding by the Brds. As depicted in Figure 3b, the nucleosomes bound separately by all three Brd family member proteins were also highly enriched with all H4 triacetylated forms when compared to whole-genome chromatin, such as the H4K5unK8acK12acK16ac (4.4- to 7.2-fold), H4K5acK8unK12acK16ac (4.7- to 7.0-fold), H4K5acK8acK12unK16ac (4.3- to 20.3-fold) and especially H4K5acK8acK12acK16un (11.3- to 44.9-fold). *t*-Tests performed with data from three independent experiments for each ChIP revealed that these fold changes are indeed significant as the *P*-values were all <1 × 10^-2 ^(Additional file 4). The finding that H4K5acK8acK12acK16un is the most enriched triacetylated H4 code further supports the hypothesis that H4K16 acetylation is not a Brd binding site. The tetracetylated H4 code, H4K5acK8acK12acK16ac, is found on 0.5% of all H4 in whole genome chromatin in HEK 293 cells; however, this form is strikingly enriched in nucleosomes bound by all three Brds - Brd2 (20.8%), Brd3 (6.8%) and Brd4 (9.3%) - and nearly undetectable in HP1-bound nucleosomes. Moreover, HP1 nucleosomes were depleted of most combinatorial forms of H4 acetylations compared to whole genome chromatin (Figure 3b).

H4K20me2 is the most abundant mark on human histone H4, found on approximately 58% of all H4 in HEK 293 cells (Additional file 2), consistent with its incorporation in heterochromatin as ≥70% of mammalian genomes are thought to be heterochromatic. H4K20me2 was enriched in HP1-bound nucleosomes and depleted in Brd nucleosomes (Figure 3b). H4K20me2 was especially depleted in Brd4-bound nucleosomes (approximately 8.5% abundance). Although this mark is extremely high in HP1-bound nucleosomes, approximately 80% for HP1β and approximately 73% for HP1α, we lack direct evidence to indicate that the HP1 chromodomains are reading this mark (Additional file 2). Similarly to H4K16ac, H4K20me2 may be part of a more complex heterochromatic histone code recognized by an HP1 complex. Additionally, as HP1 is known to bind H3K9me3, it is reasonable to speculate that the genomic location of this heterochromatic mark overlaps with H4K20me2.

### Characterization of PTMs on histone H3 from Brd- and HP1-associated nucleosomes

As indicated in Figures 2c and 3a, Brd-bound nucleosomes all have elevated levels of H3K4me1, a gene activation mark. HP1-bound nucleosomes had relatively low levels of this mark. Moreover, H3K4me2 and H3K4me3 were also elevated in the Brd-bound nucleosomes and depleted in HP1-bound nucleosomes. When compared to whole genome chromatin, nucleosomes bound by Brd2 were the most enriched in these modifications; 12.4-fold (*P*-value = 0.01) for H3K4me2 and 23.6-fold (*P*-value = 0.0009) for H3K4me3 (Figures 2c and 3a; Additional file 5). These particular PTMs occur on the nucleosomes surrounding the promoters of actively transcribed genes and are at very low levels genome-wide (≤1%).

The chromodomains of HP1α/β bind directly to silencing marks H3K9me2 and H3K9me3 [12,21]. HP1-bound nucleosomes were enriched with both these K9 methylations (Figure 2c). For these modifications, a 1.5- to 2-fold increase is significant, as these modifications are extremely abundant in whole-genome chromatin, 31% and 21%, respectively (Additional file 2). We also found that Brd-bound nucleosomes were low in H3K9me3, but have moderate levels of H3K9me2 (Figure 2c).

Another modification, H3K23me1, is also found at low abundance (0.2%), but was highly enriched on both HP1s (Figure 2c; Additional file 2). This result is not surprising as a recent study employing a histone peptide methyl-lysine array found that HP1β binds H3K23me1 in addition to H3K9me3 [22] and colocalizes with H3K9me3 and HP1β at heterochromatic loci. We believe that a subset of HP1 proteins may bind H3K23me1 via its chromodomain independent of H3K9me3 within these loci. H3K18me1 is another modification that is of very low relative abundance in whole genome chromatin (0.3%). Here we find that Brd-bound nucleosomes were highly enriched with this modification; Brd2 (5.23%), Brd3 (4.17%) and Brd4 (3.10%) (Additional file 2). H3K18 can also be acetylated by the CBP/p300 acetyltransferase, and H3K18ac localizes with H3K4me3 by ChIP at the promoters of some actively transcribed genes [3,23-25]. As depicted in Figure 2c, nucleosomes bound by Brds were enriched in H3K18ac (4.2- to 6.9-fold; *P*-values <0.001; Additional file 5) compared to bulk chromatin. This result is consistent with reports that H3K18ac localizes with H3K4me3, as Brd nucleosomes were also enriched with H3K4me3. The nucleosomes bound by the Brds also exhibited high levels of acetylation at H3K9ac, H3K14ac, and H3K23ac, whereas acetylation of these residues was reduced on HP1-associated nucleosomes (Figure 2c).

Two other abundant gene repressive PTMs are H3K27me3 and H3K27me2. Nucleosomes bound by both HP1 proteins were modestly enriched with these modifications (Figure 2c). H3K27me3 and H3K27me2 are thought to demarcate the promoter regions and bodies, respectively, of particular repressed genes. These methylations are added by the polycomb group proteins Ezh1 and Ezh2 and bound by other polycomb group chromodomain-containing CBX proteins [26,27]. Interestingly, our observation that HP1-bound nucleosomes were enriched with H3K27me2/3 suggests that there may be some overlap amongst heterochromatin bound by the HP1 and polycomb CBX chromodomain proteins. Future ChIP-seq experiments with the polycomb CBX proteins will be required to determine their genomic overlap with the HP1 proteins.

As some H3 PTMs can be observed on the same peptides, a few combinations of PTMs were also characterized (Additional file 6). We believe that there is useful combinatorial information in tandem PTMs; for example, we previously reported that the histone H3K9 euchromatic dimethytransferase G9a preferentially methylates H3 peptides that contain K14 acetylation [28]. This modification, H3K9me2K14ac, shares similar characteristics to the 'bivalent domain' (H3K4me3K27me3) found on silent genes poised for activation in pluripotent stem cells, containing PTMs with opposing epigenetic functions [29]. As mentioned previously, Brd-bound nucleosomes contained some H3K9me2, but the bulk of this modification occurred on peptides that were acetylated on the adjacent lysine (H3K9me2K14ac), the tandem marks effected by G9a (Figure 3a). This result is consistent with the observation that G9a localizes primarily to euchromatin, and that H3K9me2K14ac may also act as a combinatorial switch, similar to the bivalent domain. In contrast, H3K9me3 was depleted in Brd nucleosomes even when H3K14 was acetylated. On the other hand, there was substantial amounts of this combinatorial modification, H3K9me3K14ac, on nucleosomes bound by HP1α (13.7%) and HP1β (16.3%), which occurs on only 9.8% of the nucleosomes in the genome (Additional file 6).

Brd-bound nucleosomes also contain histone H3 enriched with other combinatorial PTMs consistent with gene activation, such as H3K9acK14ac (2.9- to 4.6-fold; *P*-values <0.038) and H3K18acK23ac (5.0- to 8.2-fold; *P*-values <0.0008; Additional file 7) and are depleted in marks that correspond with gene repression, such as H3K9me3K14un (Figure 3c). H3K36me3 is another modification associated with the bodies of actively transcribed genes. We found that Brd4-bound nucleosomes were enriched with this modification (2.2-fold), but only when the H3K27 was monomethylated (H3K27me1K36me3) (Figures 2c and 3a).

### Chromosomal locations of Brd- and HP1-bound nucleosomes (ChIP-Seq)

**Figure 4:**
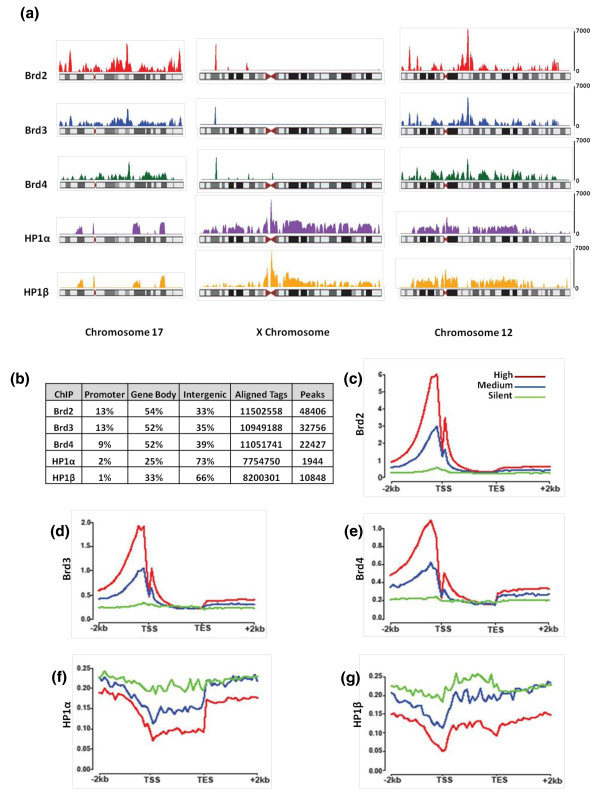
**Mapping of Brd- and HP1-bound nucleosomes to their genomic locations**. **(a) **Chromosome maps showing the density of Brd- and HP1-bound nucleosomes generated by Solexa Deep sequencing on chromosomes 17, × and 12. **(b) **Table denoting the enrichment of Brd and HP1 nucleosomes within the genome relative to the genes. **(c-g) **Graphs showing the relative enrichment of Brd and HP1 nucleosomes within genes, grouped by expression levels (high, red; medium, blue; silent, green). TES, transcription end site; TSS, transcription start site.

To determine where the histone code reading proteins genomically reside, we deep sequenced the DNA isolated in Brd- and HP1-bound nucleosomes (ChIP-seq) [4]. Figure 4a shows Brd and HP1 ChIP-seq maps for chromosomes 17, × and 12. At this level of resolution there is much overlap of the ChIP-seq maps amongst the three Brds and amongst the two HP1s. In agreement with previous reports we observed a cluster of HP1 binding that mapped within the centromere region on every chromosome [17]. HP1 binding also mapped along the entire length of the × chromosome, which is consistent with the fact that HEK293 cells are of female origin, possessing an imprinted inactive × chromosome. In contrast, Brd-binding clusters were found throughout chromosome 17 but there are only 4 predominant HP1-binding clusters, as this is a relatively small chromosome that contains many active genes, including the HOXB cluster (HOX gene clusters will be discussed later). Other chromosomes such as chromosome 12 had a more uniform distribution of Brd- and HP1-binding clusters.

At the gene level (Figure 4b), approximately 65% of Brd-binding sites were found on genes (promoter plus gene body). The remaining approximately 35% was found on intergenic regions, very often in regions flanking genes. The promoter regions as defined in this study are the +2 kb regions upstream of the transcription start sites. We observed that 9 to 13% of the Brd-binding sites were found within such promoter regions. On the other hand, HP1α and β binding sites were predominately found in intergenic regions (73% and 66%, respectively) and rarely on promoter regions of genes (1 to 2%). Complete lists of promoters bound by Brd and HP1 ChIPs can be found in Additional file 8. As shown in Figure 4c-e, patterns of Brd binding indicate that these proteins are predominately found on high and moderately expressed genes and rarely on transcriptionally silent genes. Genes were ranked based on their expression levels as determined by RNA-Seq, where high expression is defined as the top third of the genes, the middle third as moderate and the bottom third as silent. As predicted, the vast majority of genes bound by the HP1s were classified as either silent or having moderate expression (Figure 4f,g). A heatmap depicting the DNA consensus motifs enriched in the ChIPs can be found in Additional file 9 with an accompanying list of motifs (Additional file 10). We have also included lists of Gene Ontology terms enriched in the Brd ChIPs (Additional file 11).

### Brd-bound nucleosomes are associated with the promoters of HOX genes

**Figure 5:**
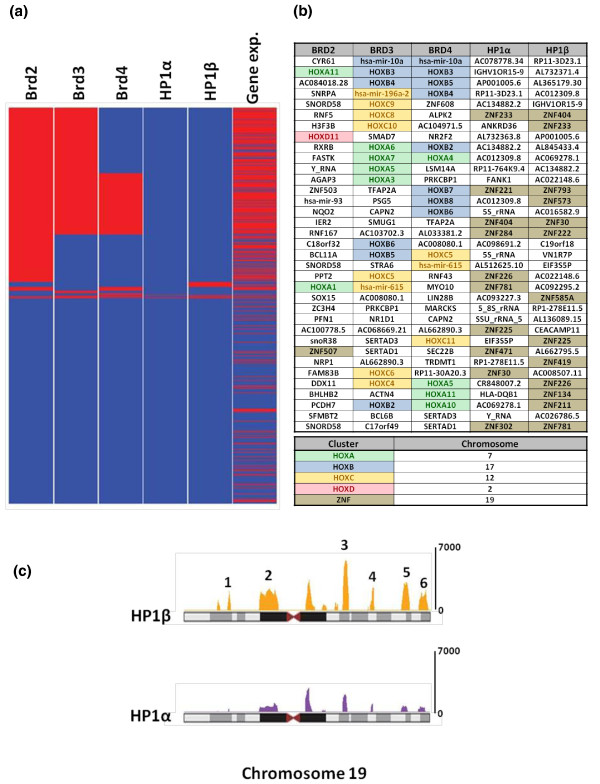
**Promoters bound by Brd- and HP1-bound nucleosomes**. **(a) **Heatmap of promoters with nucleosomes bound by the Brd and HP1 proteins. Heatmaps are arranged as clusters of promoters bound by the various ChIPs (lanes 1 to 5: bound, red; unbound, blue) and the expression of these genes from RNA-Seq is represented in lane 6 (high and medium, red; silent, blue). **(b) **Ranked order table of genes (top 35) whose promoters are bound by the Brd and HP1 proteins. Genes are ordered based on Brd/HP1 binding score within the promoter region of all annotated genes (top of the list, most bound). Genes belonging to the HOX clusters or ZNF clusters are color coded accordingly. **(c) **HP1α and HP1β binding clusters on chromosome 19. The ZNF clusters are labeled 1 to 6.

To more fully probe the genes bound by each reader, we focused on those genes whose promoters had the most significant binding. As shown in Figure 5a, genes were grouped according to the combinatorial promoter binding patterns of Brd and HP1 proteins (bound genes in red and unbound genes in blue), and expressed and non-expressed genes are shown in red and blue, respectively. We found substantial co-binding of gene promoters by the Brd proteins and a strong correlation between Brd binding and gene expression. Considering the top 500 most Brd-enriched gene promoters, we found a 54% overlap amongst Brd2 and Brd3, a 35% overlap amongst Brd3 and Brd4, and a 28% overlap between Brd2 and Brd4. As for particular genes, we observed from an unbiased ranked ordered list of bound promoters (Figure 5b) that an unusually large number of the highest ranking promoters bound by the Brd proteins were HOX genes. We have also validated the binding of endogenous Brd4 to several HOX genes (*HOXB5*, *HOXB3*, *HOXC5 *and *HOXC11*) by ChIP, using a monoclonal antibody raised against Brd4 (Additional file 12). There are four HOX gene clusters (HOXA to HOXD) in the human genome, each containing between 10 and 12 protein coding Hox (homeobox) genes as well as several microRNAs [30]. As shown in Figure 5b, when considering the top 35 promoters bound by Brd3 and Brd4, 18 and 15, respectively, are from HOX gene clusters. When we considered the top 100 scoring gene promoters bound by both Brd3 and Brd4, we found in both cases they included 15 HOX (homeobox) encoding genes and 3 HOX cluster encoded microRNAs all found within the HOXA, HOXB and HOXC clusters. As for Brd2 promoter binding, we found 9 of the top 100 ranked genes were HOX genes. Although we were surprised to find such a huge enrichment of HOX genes in Brd ChIPs, this result is in agreement with the fact that the Brd proteins are human homologs of the *Drosophila *FSH-S and FSH-L proteins, which are encoded by trithorax group genes [6]. Trithorax group genes were originally identified from *Drosophila *genetic screens as genes required for the persistent expression of HOX genes that control the development of body segments [31,32]. The differential expression of HOX genes is also essential for patterning of all vertebrate embryos and the correct sustained expression of HOX genes in different tissue types is required to maintain cells in their respective differentiated states [33]. Therefore, maintaining a specific chromatin architecture at HOX gene clusters is an elegant evolutionary solution to regulate their expression, potentially through epigenetic regulation. Other trithorax proteins that regulate HOX gene expression have been shown to be components of chromatin remodeling, histone methylation and histone acetylation complexes [34-36]. In fact, the *Drosophila *trithorax acetylation complex (TAC1) and the *Drosophila *Brd protein (FSH-S) are required for the expression of the HOX gene *Ubx *and the overexpression of FSH-S can induce the ectopic expression of several HOX genes in *Drosophila *[6,34]. These results suggest that the histone acetylation marks that are written by TAC1 are read and translated by the Brd protein FSH-S.

### HP1 bound nucleosomes are associated with the promoters of ZNF genes on chromosome 19

As stated above, the HP1s were found at the promoter regions of 1 to 2% of the genes they bind and most of these are transcriptionally silent (Figure 4b,f,g). Moreover, neither HP1 protein was found on any HOX gene promoters. We also did not observe an equal distribution amongst the chromosomes with genes whose promoters were bound by the HP1 proteins. For both HP1 proteins, approximately 30% were found on chromosome 19 and 20% were found on the × chromosome. As shown in Figure 5b, when we only considered the top 35 gene promoters bound by HP1 proteins, HP1α and HP1β bound 18 and 15 ZNF gene promoters, respectively, from chromosome 19. ZNF genes encode proteins that belong to a large superfamily of putative transcription factors (approximately 800 members in humans), many of which are thought to function as transcriptional repressors [37]. There are six clusters of ZNF genes found on chromosome 19. These clusters all span from 1 to 3 Mb of DNA each and are located along chromosome 19 as follows: cluster 1, 10 to 12 Mb; cluster 2, 19 to 23 Mb; cluster 3, 40 to 43 Mb; cluster 4, 49 to 50 Mb; cluster 5, 57 to 59 Mb; and cluster 6, 61 to 63 Mb (Figure 5c). When considering the top 100 bound promoters, we observed that HP1α bound to 37 gene promoters (23 known ZNF genes), and HP1β to 55 gene promoters (27 known ZNF genes), all located within these clusters on chromosome 19. However, we did not see complete overlap amongst the promoter binding of both HP1 proteins. While we found extensive binding of HP1β to the promoters of genes in all six clusters, HP1α was only found predominantly in clusters 3, 4 and 6, with very little binding in the other three clusters. Interestingly, the promoters of genes found in cluster 5 are extensively bound by HP1β and almost completely devoid of HP1α binding. Within this cluster, HP1β was bound to the promoters of 59 genes, 16 of which are ZNF genes, as well as a subcluster of 20 microRNA genes in the distal half of this cluster. These data are consistent with a study in 2006 that used a DNA adenine methyltransferase identification (DamID) method coupled with cDNA oligo microarrays to identify the target genes bound by HP1β [38]. In agreement with these findings, ChIP-seq experiments with antibodies directed against H3K9me3 have found distinct islands of this modification map to the ZNF clusters [4].

### Brd4 is a positive regulator of the genes it binds

**Figure 6:**
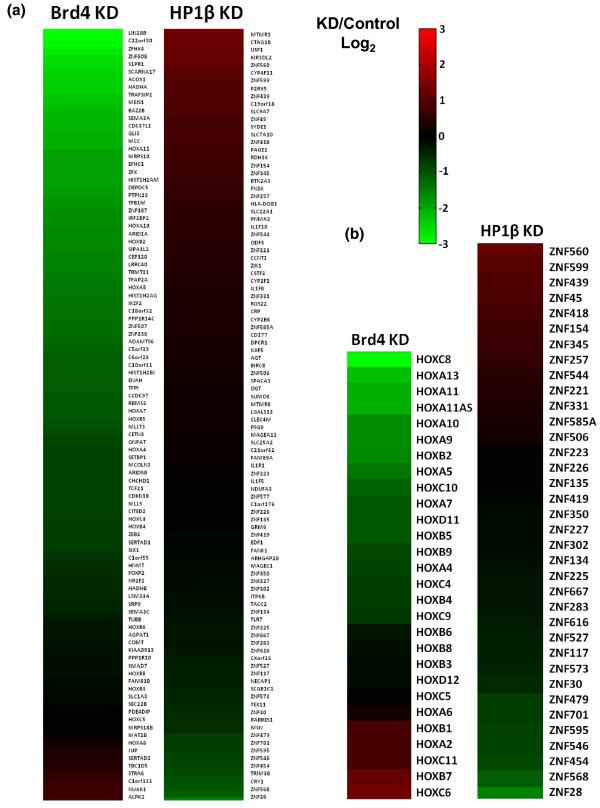
**Brd4 and HP1β regulate gene expression**. **(a) **Right panel: heatmap showing the log_2 _fold change in gene expression from 293 cells with depleted Brd4 compared to control 293 cells. Genes shown are the top 100 genes whose promoter regions were the most enriched with Brd4 bound nucleosomes. Left panel: heatmap showing the log_2 _fold change in gene expression from 293 cells with depleted HP1β compared to control 293 cells. Genes shown are the top 100 genes whose promoter regions were the most enriched with HP1β-bound nucleosomes. **(b) **Right panel: heatmap of the log_2 _fold change in expression of all HOX genes with promoters bound by Brd4 in the Brd4-depleted 293 cell line compared to control 293 cells. Left panel: heatmap of the log_2 _fold change in expression of all ZNF genes found on chromosome 19 with promoters bound by HP1β in the HP1β-depleted 293 cell line compared to control 293 cells.

In order to determine whether Brds directly regulate the expression of the genes they bind, we performed gene expression microarrays on cells depleted of Brd4 by short hairpin RNA (shRNA). We choose to knockdown Brd4 because it shared the least overall overlap of bound promoters with the other Brd ChIPs. We achieved a three-fold knockdown of Brd4 with a custom shRNA in HEK293 cells as determined by RT-PCR. The knockdown was also verified at the protein level by western blot (Additional file 13). Of the top 100 genes whose promoters were most bound by Brd4, we found three-quarters of them had at least a two-fold reduction in expression in the Brd4 shRNA cell line when compared to the control (Figure 6a, right heatmap). These results suggest that the direct binding of Brd4 to the acetylated nucleosomes associated with these genes is required for their proper expression. Only 8 of these top 100 genes showed an increase in expression upon Brd4 depletion. We speculate that these may be the result of downstream effects rather than a loss of Brd4 binding. Looking at the 28 HOX genes whose promoters were significantly bound by Brd4, we found that two-thirds of this subset of genes had reduced expression (1.5-fold or greater; Figure 6b). Only 6 of the 28 protein coding HOX genes had an increase in expression and 5 showed no significant change upon Brd4 knockdown. This result suggests that there is redundancy amongst the Brd proteins, as we found overlapping binding of the Brds on many of the HOX genes, especially between Brd3 and Brd4.

### HP1β is a negative regulator of the genes it binds

In order to determine the direct effects of HP1 binding to nucleosomes, we preformed gene expression arrays on HEK293 cells depleted of HP1β by shRNA. We chose to knockdown HP1β rather than HP1α because it was enriched on more promoters, including most of the HP1α-bound promoters. Our custom shRNA knocked down HP1β mRNA by 3.5-fold in HEK293 cells as determined by RT-PCR. The knockdown was also verified at the protein level by western blot (Additional file 13). In contrast to the effects of Brd knockdown, knockdown of HP1β resulted in at least two-fold increases expression in two-thirds of the top 100 HP1β promoter-bound genes (Figure 6a, left heatmap). These results suggest that the binding of HP1β to histone methylated nucleosomes is required for effective silencing of associated genes. We also looked at the expression of the ZNF genes found on chromosome 19 whose promoters were enriched with HP1β-bound nucleosomes (Figure 6b, left heatmap). Surprisingly, we found that the expression of only one-third of these genes significantly increased, whereas two-thirds remained the same or decreased. This result is in agreement with the aforementioned study that mapped HP1β to the ZNF clusters on chromosome 19 with the DamID method [38]. They also reported that individual depletion of HP1α/β did not lead to an appreciable change in the expression of most of the ZNF clusters. We hypothesize that this is due to the redundancy of the three HP1s (HP1α, β and γ) and attempts to circumvent this by depleting more than one at a time have been unsuccessful. Also, removing the heterochromatic binding proteins alone may not be enough to fully turn on repressed genes, as there are other steps required for gene activation, such as recruitment of transcription factors or gene activating histone-modifying enzymes or other binding proteins. This hypothesis would also be consistent with our finding that the expression of the majority of genes whose promoter regions were bound by HP1β did not change significantly when HP1β was depleted.

### Brd-bound nucleosomes are associated throughout the body of transcribed HOX genes

**Figure 7:**
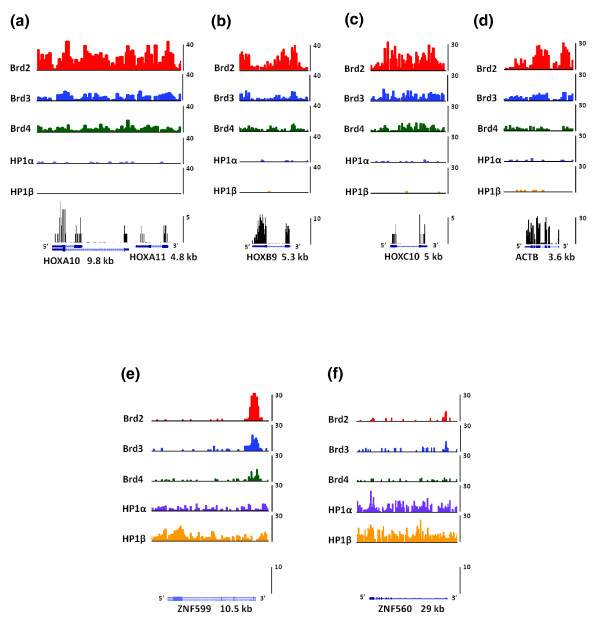
**Brd and HP1 protein nucleosomal binding patterns of Brd4 and HP1β regulated genes**. **(a) **HOXA10 and HOXA11. **(b) **HOXB9. **(c) **HOXC10. **(d) **ACTB. **(e) **ZNF599. **(f) **ZNF560. Top panels for are the ChIP-seq results depicting the Brd and HP1 binding patterns. Bottom panel is the RNA-Seq results representing the expression level of each gene.

Having selected for significantly bound genes based on Brd and HP1 promoter binding, we next looked at the binding of these proteins across the entire length of such genes. For actively transcribed genes, we examined Brd and HP1 binding across the *HOXA10*, *HOXA11*, *HOXB9*, and *HOXC10 *genes and the housekeeping gene *ACTB *(Figure 7a-d), and these genes were found to be devoid of HP1. We also found that the expression of these genes were all significantly reduced in the Brd4 knockdown cells: *HOXA10 *by 3-fold, HOXA11 by 4-fold, HOXB9 by 2-fold, HOXC10 by 2.5-fold and ACTB by 1.5-fold. The mRNA for the housekeeping gene *ACTB *is extremely abundant, so we believe that a 1.5-fold decrease in its expression is significant. Moreover, the expression of this gene increased by 1.4-fold in the HP1β kockdown cells. As shown in Figure 7a,c, binding of all three Brds was found to be evenly distributed throughout the promoter region and gene bodies of the *HOXA10*, *HOXA11 *and *HOXC10 *genes. On the *HOXB9 *gene we found a huge enrichment of Brd2 and Brd3 within the promoter region (Figure 7b). Both proteins were also bound to the body of this gene with Brd2 enrichment peaking in the later half towards the 3' region. Brd4 binding to *HOXB9 *nucleosomes was evenly distributed over the promoter region and gene body. As for the housekeeping *ACTB *gene, we found that all three Brd proteins bound within the promoter region and the gene body with Brd2 being especially enriched with the body of this gene. This is consistent with the role of Brd proteins in transcriptional elongation [8,39]. Furthermore, the binding of Brd proteins to the bodies of HOX genes is also consistent with the reported hyperacetylation of histone H4 within the bodies of HOXA genes upon activation with retinoic acid in pluripotent embryonal carcinoma cells [40].

### HP1-bound nucleosomes are associated with the bodies of transcriptionally silent ZNF genes

We next analyzed the binding patterns of HP1 proteins on the *ZNF560 *and *ZNF599 *genes (Figure 7e,f). Both genes were determined to be silent in control HEK293 cells by RNA-Seq analysis (Figure 7e,f, bottom panels). *ZNF560 *and *ZNF599 *are found in the HP1-binding clusters 3 and 6 on chromosome 19, which are significantly bound by both HP1α and HP1β. We chose these ZNF genes for further analysis as their expression was the most upregulated of all HP1-bound ZNF genes upon depletion of HP1β by shRNA (Figure 6b). As depicted in Figure 7e,f, both HP1s bound nucleosomes throughout the bodies and promoter regions of *ZNF599 *and *ZNF560*, with HP1β being slightly more enriched within the promoter and 5' region of *ZNF599*. These results, along with our array analysis, suggest that the HP1 proteins directly repress the expression of these genes. Moreover, nucleosomes found within both of these genes are relatively devoid of binding by all three Brds. Interestingly, we did observe a short cluster of Brd-bound nucleosomes at the end of the *ZNF599 *gene and continuing in the 3' direction on chromosome 19; however, this result is rather perplexing as there are no actively transcribed genes in this region.

## Discussion

Since the completion of the Human Genome Project in 2000, there have been several calls for a similar project to catalog human epigenomes. In 2008 the Alliance for the Human Epigenome and Disease (AHEAD) outlined the need for a project to provide high-resolution epigenome maps and suggested guidelines for the first pass of a human epigenome reference [41]. At the heart of these initiatives is the use of ChIPs with antibodies that recognize specific PTMs. Such studies are informative; however, ChIPs with modification-specific antibodies will only provide epigenomic maps with little knowledge on how such information is translated. Additionally, site-specific antibodies often suffer from cross-reactivity and epitope occlusion. Our less biased proteomic-genomic approaches complement such ongoing studies and shed light on how histone PTMs are translated into transcriptional outcomes.

Two different models have been proposed for the function of Brd proteins in transcription. In one model Brd4 recruits the transcription elongation factor pTefb to the transcription complex at the transcription start site, which then phosphorylates the CTD of RNA polymerase II, leading to more processive transcript elongation [39]. In the second model, Brd proteins are recruited to acetylated nucleosomes via their bromodomains and facilitate the passage of RNA polymerase II through nucleosomes by their intrinsic chaperone activity [8]. Our ChIP-seq results support both models as we have observed an enrichment of the Brd proteins at both the promoter (approximately 12%) and body (approximately 52%) of active genes. These results suggest an equal distribution of Brd proteins on promoter regions and gene bodies, as we have defined promoters regions as 2 kb upstream of the transcription start site and the average gene body is 10 to 20 kb. Moreover, the Brd-bound nucleosomes were enriched with patterns of histone PTMs found in both the promoter region and body of actively transcribed genes. ChIP-seq experiments with specific antibodies have found that the PTMs H3K9ac and H3K4me3 are associated with only a few nucleosomes at active gene promoters, whereas H3K79me1 and H4K20me1 are exclusively associated with the bodies of active genes [3,4]. When compared to whole-genome chromatin, the Brd-bound nucleosomes were enriched in all of these modifications, further suggesting that they represent a mixture of promoter and gene body nucleosomes. The Brd-bound nucleosomes were also enriched with the histone PTMs H3K14ac (approximately 55%), H3K23ac (approximately 50%), H4K12ac (approximately 50%) and H4K16ac (approximately 55%). These modifications were previously shown to be enriched on both the promoters and bodies of active genes [3,4]. Our data suggest that Brd nucleosomes are a mixture of the following: promoter bound with the predominant histone PTM patterns H3(K4me3, K9ac, K14ac, K23ac) and H4(K5ac, K12ac, K16ac), and gene body enriched with H3(K4me1, K14ac, K23ac, K79me1) and H4(K8ac, K12ac, K16ac, K20me1).

HP1-bound nucleosomes were most enriched with histones containing the repressive PTMs, H3K9me2/3 (approximately 85% combined) and H4K20me2/3 (approximately 75% combined). In addition to binding the ZNF clusters of chromosome 19, our ChIP-seq results have revealed that the HP1 proteins reside on centromeric heterochromatin and inactive × chromatin, both of which harbor these marks [3,4,17]. Also in line with our results, previous ChIP-seq experiments have colocalized these modifications to the ZNF repeat clusters on chromosome 19 [4].

Silent Hox genes are a defining hallmark of pluripotent stem cells and their expression is tightly regulated during differentiation [42]. The differential expression patterns of the Hox genes are the master regulators that direct cellular differentiation and maintain particular cell phenotypes [33]. In fact, all of the protein coding Hox genes contained bivalent domains, suggesting that they are poised for activation [29]. Moreover, another ChIP-seq study found that nearly half of all bivalent domains were bound by the polycomb PRC1 complex, which contains a chromodomain protein that binds directly to H3K27me3 [43]. Such bivalent domains were defined as containing overlapping PTMs, H3K4me3 and H3K27me3. It is yet unknown what activating or repressive marks on histone H4 are found within these bivalent domains. Future experiments will be necessary to investigate whether H4 acetylations that are bound by the Brd proteins are also present in bivalent domains. If so, it will be interesting to investigate whether Brd proteins are also bound to bivalent domain chromatin concurrent with the PRC1 binding or whether PRC1 complex is released prior to H4 acetylation and Brd binding upon gene activation. In conclusion, the proteomic and genomic techniques presented here can be used to probe the local chromatin environment bound by any chromatin-associated protein. In particular, we have examined the chromatin from genomic regions where bromodomain- or chromodomain-containing proteins are found, and quantified the histone PTMs present. These data can be used to generate hypotheses on how chromatin-associated proteins (or histone code readers) could influence the transcription of specific genes, hence yielding glimpses into how histone codes could be translated. Such experimental approaches could also be applied to any of the histone code readers in systems of biomedical importance, such as during stem cell differentiation or the metastatic progression of cancers.

## Materials and methods

### Chromatin immunoprecipitation

Mononucleosomes were purified after (micrococcal nuclease) Mnase digestion with FLAG-Brd or FLAG-HP1 proteins as described in [8] from HEK293 cells. FLAG-HP1 vectors were described in [44]. All proteins were generated by transient expression as previously described [8,44], and expression assessed by western blots as shown in Additional file 1. The quality of the ChIPs was analyzed by 17% SDS-PAGE and Coommassie blue staining. The histone protein ChIP inputs and elutions were propionyl derivatized with either 'light' *d_0_*- or 'heavy' *d_10_*-propionic anhydride, trypsinized and prepared for mass spectrometry as previously described in [18] with minor adjustments. 2-Propanol was substituted for methanol during the derivatization reaction as we have found that methanol can modify peptides at low pH.

### Liquid chromatography-mass spectrometry-based proteomics

Histone peptides were separated by reverse phase nanospray liquid chromatography on C18 resin with an Agilent 1200 series HPLC. Mass spectrometry was performed on a LTQ-Orbitrap mass spectrometer (ThermoFisher Scientific Inc. Waltham, MA, USA as previously described [18].

### Mass spectrometry data analysis

The LC-MS/MS data sets were analyzed using in-house software. Briefly, the characterization of the histone PTMs was performed using an optimization-based algorithmic framework that simultaneously utilizes mass spectrometry, MS/MS and chromatographic information to identify and quantify the PTMs on the histone peptides. The method automatically considers multiple charge states of all modified peptides and is able to resolve mixed tandem mass spectra resulting from co-eluting and isobaric modified peptides using a superposition method, as previously described [45]. The statistical significance of the relative abundance of histone modifications in various ChIPs as compared to the relative abundance of genomic histone PTMs was calculated using a two-tailed, unpaired Student's *t*-test, and the associated *P*-values were generated using Microsoft Excel. Mass spectrometric data were converted from RAW format into mzXML format using ReAdW. The mzXML files were deposited into the Tranche repository and are available for download from the Proteome Commons [46].

### Preparation of DNA for deep sequencing analysis

Nucleosomal DNAs were treated with alkaline phosphatase to remove the 3' phosphates left after MNase digestion and purified by phenol extraction. The DNA fragments (approximately 1 μg) were further prepared using the Solexa^® ^Illumina library kit directly following the manufacturer's protocol. The purified DNA was used directly for cluster generation and sequencing using a Solexa 1G Genome Analyzer (Illumina).

### Preparation of RNA for RNA-Seq analysis

The raw sequence data for the RNA-Seq in HEK293 cells were obtained from [47] (Gene Expression Omnibus sample ID GSM301568). The RNA preparation and sequencing protocols for this RNA-Seq data can be found in [47].

### Deep sequencing analysis

The raw sequence reads were aligned to the hg18 human genome using the Bowtie short read aligner [48]. To avoid possible PCR artifacts, we kept at most one uniquely aligned read at each genomic location per strand. To determine the number of ChIP-Seq sequencing reads in a genomic interval, we shifted the locations of forward and reverse strand tags by 75 bp, half the size of the nucleosomal DNA.

For the chromosomal binding patterns of Brd and HP1 proteins (Figures 4a and 5c), reads were counted in sliding windows of 1 Mb with a step size of 100 kb. The read counts were normalized to the sequencing depth of 5 million non-redundant reads. The signal level in each 1-Mb window was computed as the difference between the normalized read counts in the window in the sample and the control libraries (N_sample _- N_control_) with the negative values set to zero. A clustering approach described in [49] was used to identify Brd and HP1 protein-enriched domains ('islands') in the human genome from the ChIP-Seq data. The percentage overlap (Figure 4b) of the Brd and HP1 islands with the promoter, the gene-body and the inter-genic regions were determined using the gene annotations from the Ensembl database.

The sequence read density (per 100-bp window and 5 million non-redundant reads) was determined along the gene body and in the upstream/downstream 2-kb regions in the sample and control libraries. The signal level in each window was computed as the difference between the read densities in the sample and control libraries, with the negative values set to zero. Genes were ranked based on their expression levels estimated as reads per kilobase per million mapped reads (RPKM [50] and stratified into three expression groups: the 'silent' group (the bottom 30% genes in the ranked list), the 'high' group (the top 30% genes), and the 'medium' group (the remaining genes). The mean signal levels over all genes in each group were determined and are displayed as gene-body plots in Figure 4c-g.

The patterns of the combinatorial binding of Brd and HP1 proteins to the gene promoters and their correlation to the gene expression levels were determined as follows. A gene promoter, defined as a 2-kb region upstream of the transcription start site, is deemed bound by the protein if the island (determined as described above) overlaps the promoter. Genes with a particular combinatorial promoter-binding pattern of the five proteins (such as 'red-red-red-blue-blue' in Figure 5a) were grouped. The groups were then ranked based on the number of their member genes and displayed in the descending order in Figure 5a (except for the most populous group, 'blue-blue-blue-blue-blue', displayed at the bottom). The expression status of each gene is indicated as expressed ('red') if its RPKM value is at least 1 and not expressed ('blue') otherwise. The UCSC genome browser [51] bedGraph files displayed in Figure 7a-f were generated as follows. For the ChIP-Seq data, the 75-bp-shifted non-redundant reads were counted in 200-bp tiling windows along the genome and the read counts were normalized to a library size of 5 million non-redundant uniquely mapped reads in each library. For the RNA-Seq data, the centers of the reads were counted in 20-bp tiling windows along the human genome. We deposited raw and analyzed ChIP-Seq and microarray data in the Gene Expression Omnibus [52].

### Brd4 and HP1β knockdown cells

HEK293 cells were infected with lentiviral MISSION^® ^(Sigma-Aldrich Corporation, St. Louis, MO, USA) shRNA vectors (pLKO.1-puro) harboring shRNAs to Brd4 (clone TRCN0000062223), HP1β (clone TRCN0000021427) or control (empty pLKO.1-puro vector). After 72 hours the infected cells were selected with puromycin according to the manufacturer's protocols (Sigma Aldrich). Knockdowns of target RNAs were analyzed by qRT-PCR. For qRT-PCR, RNA from cells was purified with an RNeasy kit (Qiagen) and prepared with a cDNA synthesis kit (Invitrogen Carlsbad, CA, USA). qRT-PCR reactions were performed with SYBR FAST qPCR supermix (KAPA Biosystems Woburn, MA, USA).

### Microarray analysis

RNA was extracted and purified with an RNeasy kit (Qiagen) from Brd4, HP1β and control shRNA knockdown HEK293 cells. For each sample 400 ng RNA was linearly amplified and labeled with Cy5-CTP using Low RNA Linear Amplification reagents (Agilent Technologies Santa Clara, CA, USA). Reference RNA from wild-type HEK293 cells was purified, amplified and labeled with Cy3 in the same manner. Equal quantities of the Cy5- and Cy3-labeled cRNAs were mixed and competitively hybridized to human GE 4x44K microarrays (Agilent, G4112F) for 18 h using an Agilent hybridization kit. Slides were washed according to the manufacturer's protocols and scanned using an Agilent G2565BA scanner. Raw image data were extracted and subjected to standard background subtraction and linear and lowess normalization using Agilent Feature extraction software v9.5. Heatmaps were generated using MATLAB^® ^software (MathWorks Natick, MA, USA). More detailed methods are described in Additional file 14.

## Abbreviations

bp: base pair; ChIP: chromatin immunoprecipitation; ChIP-seq: chromatin immunoprecipitation-high throughput deep sequencing; DamID: DNA adenine methyltransferase identification; nanoLC-MS/MS: nano scale liquid chromatography-tandem mass spectrometry; PCR: polymerase chain reaction; PHD: plant homeodomain; PTM: post-translational modification; qMS: quantitative mass spectrometry; RNA-Seq: RNA-high-throughput deep sequencing; RPKM: reads per kilobase per million mapped reads; shRNA: short hairpin RNA.

## Competing interests

The authors declare that they have no competing interests.

## Authors' contributions

GL performed most of the experimental work. IC carried out the analysis of the deep sequencing data. PAD helped analyze the mass spectrometry data. MAB helped with the analysis of microarray data. BMZ assisted with experimental work. KZ and BAG supervised and participated in the design and coordination of the project. GL and BAG wrote the manuscript. All authors read and approved the final manuscript.

## Supplementary Material

Additional file 1 **Western blots of whole cell extracts from cell lines expressing FLAG-Brd4, FLAG-HP1β and control cell line (empty vector)**. Blots were probed with anti-Brd4 (mAb Epitomics, 5716 Burlingame, CA, USA), anti-HP1β (pAb Cell Signaling Technology 2613 Danvers, MA, USA) and β-actin control (mAb Santa Cruz, sc-81178 Santa Cruz, CA, USA). Click here for file

Additional file 2 **Table of relative PTM abundances determined by quantitative mass spectrometry on histones H3 and H4 averaged from three independent ChIP experiments with each Brd and HP1 protein and data from three experiments with HEK293 genomic chromatin**. Click here for file

Additional file 3 **Table of relative PTM abundances determined by quantitative mass spectrometry on the histone H4 peptide (amino acids 4 to 17) averaged from three independent ChIP experiments with each Brd and HP1 protein and data from three experiments with HEK293 genomic chromatin**. Click here for file

Additional file 4 ***P*-values from *t*-tests performed on the fold changes (ChIP/Genomic) from the histone H4 data presented in **Additional file 3. *t*-Tests were performed with data from three independent ChIP experiments for each Brd and HP1 protein and data from three experiments with HEK293 genomic chromatin. *P*-values were adjusted using the Benjamini-Hochberg correction method to control the false discovery rate (FDR). Click here for file

Additional file 5 ***P*-values from *t*-tests performed on the fold changes (ChIP/Genomic) from the histone data presented in **Additional file 2. *t*-Tests were performed with data from three independent ChIP experiments for each Brd and HP1 protein and data from three experiments with HEK293 genomic chromatin. *P*-values were adjusted using the Benjamini-Hochberg correction method to control the false discovery rate (FDR). Click here for file

Additional file 6 **Table of relative combinatorial PTM abundances determined by quantitative mass spectrometry on the histone H3 peptides (amino acids 9 to 17), (amino acids 18 to 26) and (amino acids 27 to 40) averaged from three independent ChIP experiments with each Brd and HP1 protein and data from three experiments with HEK293 genomic chromatin**. Click here for file

Additional file 7 ***P*-values from *t*-tests performed on the fold changes (ChIP/Genomic) from the histone H3 data presented in **Additional file 6. *t*-Tests were performed with data from three independent ChIP experiments for each Brd and HP1 protein and data from three experiments with HEK293 genomic chromatin. *P*-values were adjusted using the Benjamini-Hochberg correction method to control the false discovery rate (FDR). Click here for file

Additional file 8 **Spreadsheets of all promoters bound by the Brd and HP1 proteins**. Promoters are ranked by *P*-values. Click here for file

Additional file 9 **Heatmap of motifs enriched in the HP1 and Brd ChIPs**. Lists of consensus sequences (motifs) are found in the matrix used to create the heatmap (Additional file 10) Click here for file

Additional file 10 **Spreadsheets containing matrix used to create the heatmap of motifs enriched in Brd and HP1 ChIPs **(Additional file 9). Click here for file

Additional file 11 **Spreadsheets containing Gene Ontology terms enriched in Brd and HP1 ChIPs. Gene Ontology terms are ranked by false discovery rates (FDRs)**. Click here for file

Additional file 12 **Products from PCR reactions were run on 2% agarose gels stained with ethidium bromide and visualized on a Gel Doc XR system (BioRad^® ^Hercules, CA, USA)**. One half of each PCR reaction was loaded. Gel is labeled corresponding to the templates used for the PCR reactions: control ChIP (beads alone), Brd4 ChIP and ChIP input DNA. Click here for file

Additional file 13 **Western blots of whole cell extracts from Brd4 shRNA knockdown, HP1β shRNA knockdown and control shRNA knockdown cell lines**. Blots were probed with anti-Brd4 (mAb Epitomics, 5716), anti-HP1β (pAb Cell Signaling Technology 2613) and β-actin control (mAb Santa Cruz, sc-81178). Click here for file

Additional file 14 **Supplemental Materials and methods**. Click here for file
