# Supplementary figures and images for: Proteogenomic characterization and mapping of nucleosomes decoded by Brd and HP1 proteins
Source: Genome Biol. 2012 Aug 16;13(8):R68. doi: 10.1186/gb-2012-13-8-r68 (PMC3491368; doi:10.1186/gb-2012-13-8-r68)

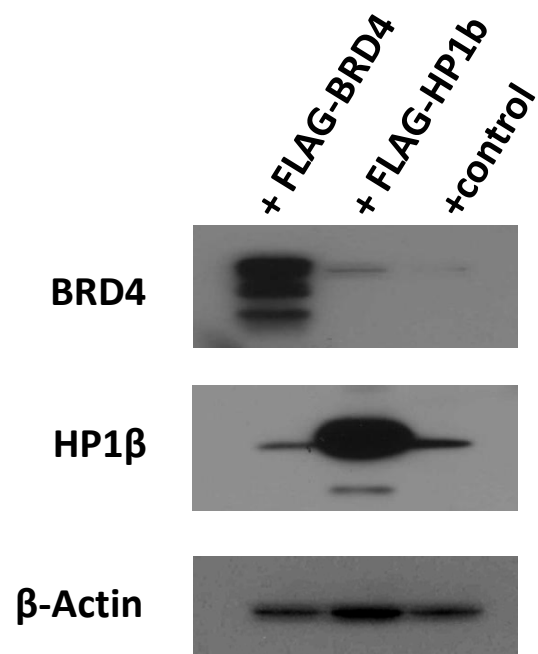

Supplement: Additional file 1 — Western blots of whole cell extracts from cell lines expressing FLAG-Brd4, FLAG-HP1β and control cell line (empty vector). Blots were probed with anti-Brd4 (mAb Epitomics, 5716 Burlingame, CA, USA), anti-HP1β (pAb Cell Signaling Technology 2613 Danvers, MA, USA) and β-actin control (mAb Santa Cruz, sc-81178 Santa Cruz, CA, USA). [file gb-2012-13-8-r68-S1.PDF]

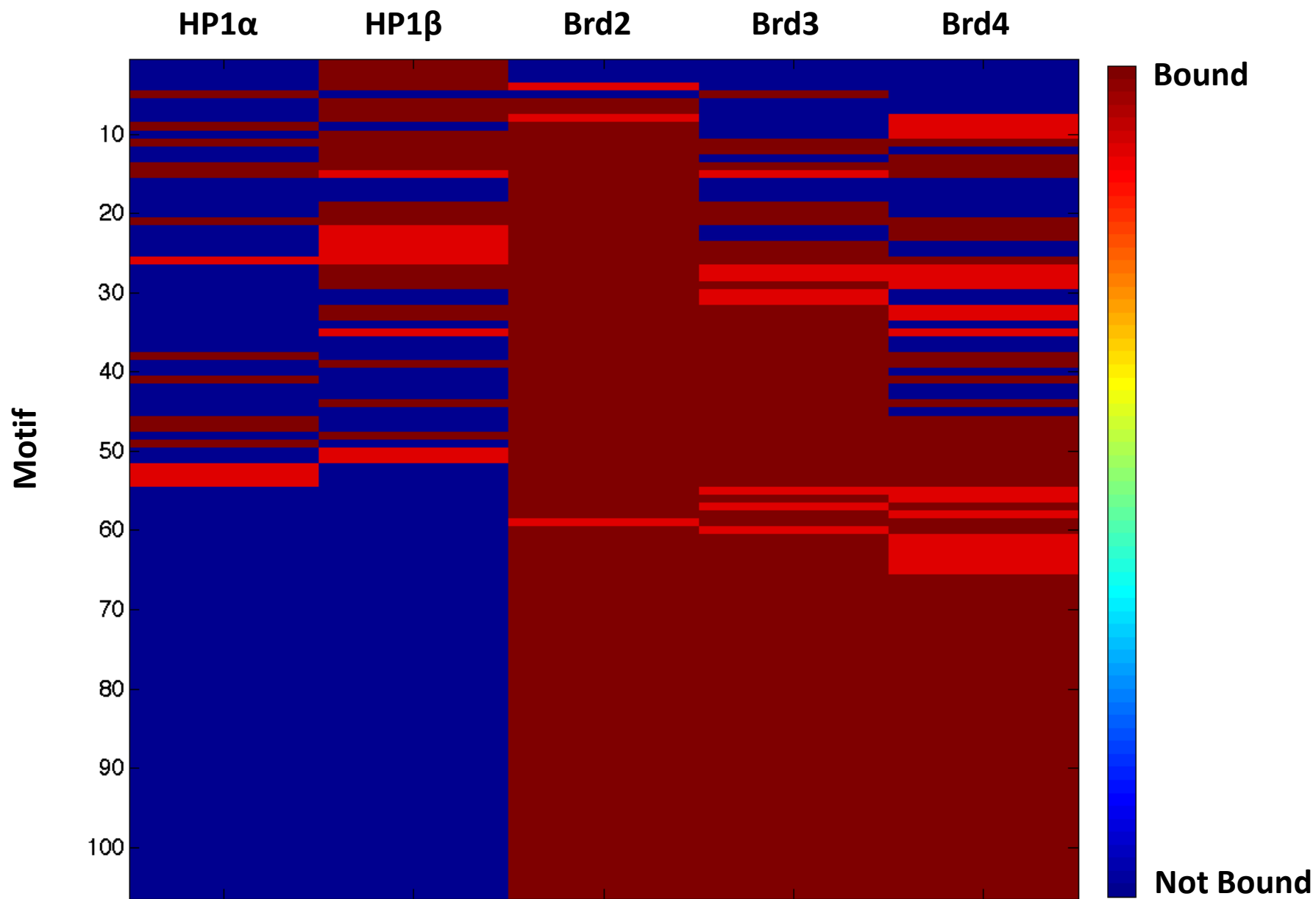

Supplement: Additional file 9 — Heatmap of motifs enriched in the HP1 and Brd ChIPs. Lists of consensus sequences (motifs) are found in the matrix used to create the heatmap (Additional file 10) [file gb-2012-13-8-r68-S9.PDF]

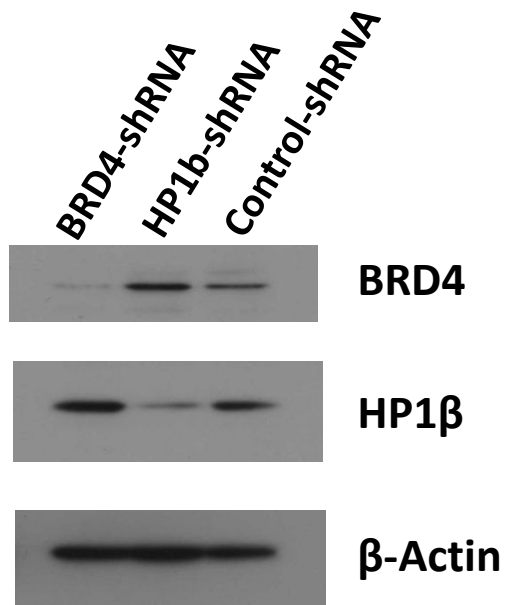

Supplement: Additional file 13 — Western blots of whole cell extracts from Brd4 shRNA knockdown, HP1β shRNA knockdown and control shRNA knockdown cell lines. Blots were probed with anti-Brd4 (mAb Epitomics, 5716), anti-HP1β (pAb Cell Signaling Technology 2613) and β-actin control (mAb Santa Cruz, sc-81178). [file gb-2012-13-8-r68-S13.PDF]
