# Supplementary material for: Proteogenomic characterization and mapping of nucleosomes decoded by Brd and HP1 proteins
Source: Genome Biol. 2012 Aug 16;13(8):R68. doi: 10.1186/gb-2012-13-8-r68 (PMC3491368; doi:10.1186/gb-2012-13-8-r68)
Supplement: Additional file 2 — Table of relative PTM abundances determined by quantitative mass spectrometry on histones H3 and H4 averaged from three independent ChIP experiments with each Brd and HP1 protein and data from three experiments with HEK293 genomic chromatin. [file gb-2012-13-8-r68-S2.PDF]

Modification	Brd2	Brd3	Brd4	HP1α	HP1β	Genomic
H3K4me1	34.73% ± 0.73%	21.83% ± 2.33%	24.79% ± 3.46%	4.37% ± 1.75%	13.75% ± 1.52%	14.17% ± 0.54%
H3K4me2	8.68% ± 2.47%	3.37% ± 1.13%	2.48% ± 0.25%	0.03% ± 0.01%	0.02% ± 0.01%	0.70% ± 0.17%
H3K4me3	3.20% ± 0.36%	0.94% ± 0.20%	0.53% ± 0.12%	0.01% ± 0.00%	0.01% ± 0.01%	0.14% ± 0.01%
H3K9me1	27.80% ± 2.62%	23.81% ± 2.41%	23.27% ± 1.84%	9.62% ± 0.98%	7.74% ± 1.02%	21.85% ± 2.88%
H3K9me2	25.13% ± 1.17%	30.45% ± 2.97%	35.13% ± 2.37%	47.09% ± 0.44%	43.46% ± 1.63%	31.08% ± 2.01%
H3K9me3	8.53% ± 1.05%	12.96% ± 0.86%	12.12% ± 0.58%	35.64% ± 0.27%	45.07% ± 2.53%	21.92% ± 1.35%
H3K9ac	8.43% ± 0.87%	5.94% ± 0.39%	4.46% ± 1.01%	0.48% ± 0.13%	0.59% ± 0.28%	1.70% ± 0.48%
H3K14ac	58.20% ± 2.40%	51.57% ± 1.86%	56.34% ± 0.55%	41.17% ± 2.59%	36.58% ± 3.20%	44.70% ± 1.21%
H3K18me1	5.23% ± 1.78%	4.17% ± 0.61%	3.10% ± 0.67%	0.25% ± 0.09%	0.31% ± 0.06%	0.29% ± 0.05%
H3K18ac	14.35% ± 1.76%	9.38% ± 1.04%	15.51% ± 1.40%	3.23% ± 0.22%	3.21% ± 0.76%	2.26% ± 0.20%
H3K23me1	0.61% ± 0.34%	0.41% ± 0.20%	0.28% ± 0.15%	5.13% ± 1.65%	4.37% ± 0.43%	0.20% ± 0.03%
H3K23ac	50.12% ± 3.45%	49.82% ± 1.34%	53.86% ± 2.13%	25.97% ± 2.02%	27.83% ± 0.72%	37.22% ± 3.61%
H3K27me1	40.81% ± 1.48%	27.81% ± 5.52%	23.27% ± 4.11%	13.44% ± 2.79%	14.11% ± 1.63%	35.98% ± 1.24%
H3K27me2	33.83% ± 2.97%	47.03% ± 4.27%	55.94% ± 5.39%	54.97% ± 3.25%	56.34% ± 2.45%	34.87% ± 0.12%
H3K27me3	10.88% ± 1.16%	16.69% ± 2.61%	16.28% ± 1.60%	28.92% ± 1.96%	26.04% ± 3.37%	12.74% ± 0.43%
H3K27ac	0.04% ± 0.01%	0.01% ± 0.01%	0.04% ± 0.02%	0.01% ± 0.01%	0.01% ± 0.00%	0.06% ± 0.01%
H3K36me1	35.28% ± 3.64%	36.80% ± 4.65%	34.50% ± 1.21%	41.69% ± 3.73%	35.34% ± 2.92%	29.00% ± 3.29%
H3K36me2	16.05% ± 3.09%	29.76% ± 4.61%	23.80% ± 1.53%	15.56% ± 1.63%	11.18% ± 2.28%	24.93% ± 3.21%
H3K36me3	2.02% ± 0.47%	3.54% ± 2.85%	7.07% ± 1.89%	2.45% ± 0.78%	2.21% ± 0.31%	3.30% ± 0.62%
H3K79me1	30.79% ± 0.28%	26.72% ± 1.41%	35.62% ± 2.40%	17.56% ± 4.36%	19.17% ± 3.12%	18.78% ± 1.18%
H3K79me2	3.62% ± 0.33%	3.04% ± 0.04%	1.61% ± 0.48%	0.62% ± 0.28%	4.96% ± 1.64%	1.89% ± 0.15%
H4K5ac	46.03% ± 1.82%	26.26% ± 0.59%	34.52% ± 2.88%	1.53% ± 0.38%	4.98% ± 1.01%	7.29% ± 0.54%
H4K8ac	45.48% ± 2.22%	21.52% ± 0.65%	31.38% ± 2.75%	1.94% ± 0.31%	2.93% ± 0.64%	5.21% ± 0.56%
H4K12ac	62.28% ± 2.84%	42.81% ± 4.40%	62.42% ± 3.36%	8.48% ± 1.06%	12.18% ± 2.13%	18.71% ± 1.76%
H4K16ac	62.20% ± 3.22%	49.94% ± 2.09%	58.91% ± 4.32%	17.44% ± 3.80%	26.18% ± 2.39%	41.30% ± 2.89%
H4K20me1	31.38% ± 3.25%	43.66% ± 0.79%	41.98% ± 4.70%	15.98% ± 1.75%	14.99% ± 1.83%	24.92% ± 1.12%
H4K20me2	35.17% ± 2.33%	25.57% ± 0.34%	8.49% ± 1.76%	72.95% ± 5.17%	79.75% ± 1.52%	57.88% ± 1.34%
H4K20me3	0.33% ± 0.14%	0.41% ± 0.08%	0.08% ± 0.04%	1.59% ± 0.31%	2.61% ± 0.67%	1.21% ± 0.07%
