# Supplementary material for: Proteogenomic characterization and mapping of nucleosomes decoded by Brd and HP1 proteins
Source: Genome Biol. 2012 Aug 16;13(8):R68. doi: 10.1186/gb-2012-13-8-r68 (PMC3491368; doi:10.1186/gb-2012-13-8-r68)
Supplement: Additional file 3 — Table of relative PTM abundances determined by quantitative mass spectrometry on the histone H4 peptide (amino acids 4 to 17) averaged from three independent ChIP experiments with each Brd and HP1 protein and data from three experiments with HEK293 genomic chromatin. [file gb-2012-13-8-r68-S3.PDF]

H4 Peptide	Brd2	Brd3	Brd4	HP1α	HP1β	Genomic
GKGGKGLGKGGAKR (4-17)						
H4K5unK8unK12unK16un	15.12% ± 1.49%	29.35% ± 1.51%	15.58% ± 2.86%	74.12% ± 3.46%	63.31% ± 3.87%	47.46% ± 2.08%
H4K5unK8unK12unK16ac	12.12% ± 0.64%	18.06% ± 2.39%	11.08% ± 1.07%	15.73% ± 3.47%	19.51% ± 1.83%	29.14% ± 3.36%
H4K5unK8unK12acK16un	5.92% ± 1.00%	8.43% ± 2.42%	8.48% ± 1.33%	5.74% ± 1.19%	5.07% ± 1.53%	4.36% ± 1.10%
H4K5unK8acK12unK16un	1.01% ± 0.55%	1.67% ± 0.63%	1.08% ± 0.31%	0.25% ± 0.11%	0.44% ± 0.10%	1.21% ± 0.27%
H4K5acK8unK12unK16un	1.24% ± 0.23%	2.42% ± 0.83%	2.62% ± 0.51%	0.94% ± 0.35%	2.61% ± 0.82%	1.90% ± 0.24%
H4K5unK8unK12acK16ac	8.27% ± 0.51%	8.03% ± 0.73%	16.06% ± 0.83%	1.14% ± 0.32%	4.74% ± 1.36%	7.54% ± 0.76%
H4K5unK8acK12unK16ac	0.88% ± 0.18%	1.55% ± 0.55%	1.58% ± 0.44%	0.23% ± 0.11%	0.22% ± 0.09%	0.22% ± 0.10%
H4K5unK8acK12acK16un	3.14% ± 0.66%	1.33% ± 0.26%	3.02% ± 0.79%	1.18% ± 0.15%	1.65% ± 0.55%	1.57% ± 0.36%
H4K5acK8unK12unK16ac	0.85% ± 0.11%	0.70% ± 0.17%	0.87% ± 0.25%	0.11% ± 0.07%	1.36% ± 0.04%	1.01% ± 0.17%
H4K5acK8unK12acK16un	0.71% ± 0.09%	4.16% ± 1.54%	3.02% ± 0.44%	0.19% ± 0.08%	0.40% ± 0.13%	1.83% ± 0.11%
H4K5acK8acK12unK16un	5.03% ± 1.02%	1.27% ± 0.37%	4.31% ± 1.07%	0.12% ± 0.01%	0.29% ± 0.11%	0.25% ± 0.05%
H4K5unK8acK12acK16ac	7.51% ± 0.56%	5.32% ± 0.40%	8.60% ± 1.36%	0.09% ± 0.03%	0.08% ± 0.06%	1.20% ± 0.03%
H4K5acK8unK12acK16ac	10.29% ± 0.26%	7.33% ± 1.47%	10.90% ± 1.61%	0.10% ± 0.03%	0.07% ± 0.03%	1.55% ± 0.12%
H4K5acK8acK12unK16ac	1.47% ± 0.46%	2.16% ± 0.27%	0.46% ± 0.05%	0.02% ± 0.01%	0.07% ± 0.03%	0.11% ± 0.04%
H4K5acK8acK12acK16un	5.63% ± 0.51%	1.41% ± 0.36%	3.00% ± 0.93%	0.02% ± 0.01%	0.03% ± 0.02%	0.13% ± 0.06%
H4K5acK8acK12acK16ac	20.81% ± 2.85%	6.80% ± 0.75%	9.35% ± 1.70%	0.03% ± 0.01%	0.13% ± 0.01%	0.53% ± 0.05%
