# Supplementary material for: Proteogenomic characterization and mapping of nucleosomes decoded by Brd and HP1 proteins
Source: Genome Biol. 2012 Aug 16;13(8):R68. doi: 10.1186/gb-2012-13-8-r68 (PMC3491368; doi:10.1186/gb-2012-13-8-r68)
Supplement: Additional file 4 — P-values from t-tests performed on the fold changes (ChIP/Genomic) from the histone H4 data presented in Additional file 3. t-Tests were performed with data from three independent ChIP experiments for each Brd and HP1 protein and data from three experiments with HEK293 genomic chromatin. P-values were adjusted using the Benjamini-Hochberg correction method to control the false discovery rate (FDR). [file gb-2012-13-8-r68-S4.PDF]

Modification	Brd2		Brd3		Brd4		HP1α		HP1β	
GKGGKGLGKGGAKR (4-17)	p-value	Adjusted	p-value	Adjusted	p-value	Adjusted	p-value	Adjusted	p-value	Adjusted
H4K5unK8unK12unK16un	0.000026	0.000561	0.000258	0.001360	0.000098	0.000956	0.000332	0.001537	0.003336	0.007714
H4K5unK8unK12unK16ac	0.001000	0.003333	0.009630	0.017381	0.000897	0.003191	0.008589	0.015733	0.012116	0.020470
H4K5unK8unK12acK16un	0.143366	0.175648	0.056930	0.076293	0.014431	0.023625	0.216251	0.254010	0.554039	0.594187
H4K5unK8acK12unK16un	0.604877	0.635809	0.307892	0.349448	0.612648	0.638535	0.005024	0.010453	0.010691	0.019000
H4K5acK8unK12unK16un	0.025249	0.037519	0.350197	0.395039	0.090805	0.114280	0.017375	0.026899	0.218446	0.255775
H4K5unK8unK12acK16ac	0.239513	0.276937	0.467514	0.514821	0.000196	0.001228	0.000174	0.001148	0.035802	0.049988
H4K5unK8acK12unK16ac	0.004976	0.010453	0.014820	0.024050	0.006320	0.012504	0.943521	0.946078	0.994982	0.994982
H4K5unK8acK12acK16un	0.022666	0.034370	0.394102	0.441872	0.044883	0.062198	0.150227	0.182843	0.844974	0.856549
H4K5acK8unK12unK16ac	0.247736	0.285552	0.090444	0.114280	0.486062	0.533658	0.001138	0.003728	0.026177	0.038283
H4K5acK8unK12acK16un	0.000164	0.001141	0.058619	0.078018	0.010807	0.019041	0.000034	0.000630	0.000136	0.001045
H4K5acK8acK12unK16un	0.001237	0.003845	0.009126	0.016634	0.002763	0.006638	0.011345	0.019691	0.563922	0.603038
H4K5unK8acK12acK16ac	0.000041	0.000695	0.000058	0.000822	0.000714	0.002694	0.000001	0.000163	0.000006	0.000254
H4K5acK8unK12acK16ac	0.000001	0.000163	0.002428	0.006028	0.000553	0.002301	0.000034	0.000630	0.000032	0.000630
H4K5acK8acK12unK16ac	0.007182	0.013768	0.000188	0.001200	0.000583	0.002395	0.024255	0.036481	0.257646	0.296053
H4K5acK8acK12acK16un	0.000050	0.000750	0.003667	0.008222	0.005900	0.011800	0.035037	0.049104	0.061224	0.080615
H4K5acK8acK12acK16ac	0.000247	0.001346	0.000130	0.001027	0.000841	0.003052	0.000070	0.000873	0.000169	0.001141
