# Supplementary material for: Proteogenomic characterization and mapping of nucleosomes decoded by Brd and HP1 proteins
Source: Genome Biol. 2012 Aug 16;13(8):R68. doi: 10.1186/gb-2012-13-8-r68 (PMC3491368; doi:10.1186/gb-2012-13-8-r68)
Supplement: Additional file 5 — P-values from t-tests performed on the fold changes (ChIP/Genomic) from the histone data presented in Additional file 2. t-Tests were performed with data from three independent ChIP experiments for each Brd and HP1 protein and data from three experiments with HEK293 genomic chromatin. P-values were adjusted using the Benjamini-Hochberg correction method to control the false discovery rate (FDR). [file gb-2012-13-8-r68-S5.PDF]

Modification	Brd2		Brd3		Brd4		HP1α		HP1β	
	p-value	Adjusted	p-value	Adjusted	p-value	Adjusted	p-value	Adjusted	p-value	Adjusted
H3K4me1	0.000003	0.000163	0.005192	0.010555	0.006271	0.012475	0.000751	0.002778	0.675802	0.692315
H3K4me2	0.005029	0.010453	0.015557	0.024918	0.000510	0.002169	0.002386	0.006006	0.002287	0.005959
H3K4me3	0.000119	0.000990	0.002374	0.006006	0.004630	0.009901	0.000084	0.000873	0.000229	0.001301
H3K9me1	0.057116	0.076293	0.418268	0.463351	0.511526	0.558303	0.002234	0.005861	0.001323	0.003981
H3K9me2	0.011407	0.019691	0.776277	0.789073	0.087131	0.110786	0.000177	0.001148	0.001169	0.003760
H3K9me3	0.000170	0.001141	0.000630	0.002508	0.000319	0.001493	0.000066	0.000873	0.000152	0.001088
H3K9ac	0.000298	0.001449	0.000285	0.001444	0.012900	0.021500	0.013102	0.021738	0.025557	0.037825
H3K14ac	0.000955	0.003333	0.005854	0.011772	0.000109	0.000966	0.098981	0.123727	0.014704	0.023967
H3K18me1	0.008558	0.015733	0.000379	0.001689	0.001874	0.005097	0.599058	0.631485	0.643819	0.667264
H3K18ac	0.000295	0.001449	0.000310	0.001469	0.000083	0.000873	0.004967	0.010453	0.104603	0.129652
H3K23me1	0.105124	0.129652	0.153596	0.185721	0.413395	0.459328	0.006671	0.012923	0.000076	0.000873
H3K23ac	0.011038	0.019356	0.004798	0.010202	0.002342	0.006006	0.009239	0.016758	0.011549	0.019691
H3K27me1	0.012265	0.020628	0.066661	0.087155	0.006839	0.013179	0.000216	0.001257	0.000051	0.000750
H3K27me2	0.576005	0.610664	0.007883	0.014730	0.002484	0.006088	0.000434	0.001866	0.000110	0.000966
H3K27me3	0.059257	0.078584	0.061001	0.080608	0.021056	0.032061	0.000152	0.001088	0.002471	0.006088
H3K27ac	0.084275	0.107523	0.005133	0.010505	0.298984	0.340382	0.002835	0.006724	0.002369	0.006006
H3K36me1	0.090806	0.114280	0.076717	0.098560	0.053150	0.072300	0.011510	0.019691	0.067125	0.087452
H3K36me2	0.025936	0.038181	0.211075	0.250314	0.609308	0.636848	0.010733	0.019000	0.003765	0.008343
H3K36me3	0.048095	0.065665	0.893230	0.898085	0.030422	0.043647	0.214002	0.252168	0.054531	0.073637
H3K79me1	0.000069	0.000873	0.001710	0.004827	0.000402	0.001759	0.664311	0.683294	0.850228	0.859520
H3K79me2	0.001234	0.003845	0.000235	0.001301	0.382190	0.429818	0.002407	0.006018	0.032422	0.045787
H4K5ac	0.000004	0.000202	0.000002	0.000163	0.000087	0.000873	0.000112	0.000967	0.024966	0.037247
H4K8ac	0.000007	0.000254	0.000005	0.000230	0.000086	0.000873	0.000895	0.003191	0.009771	0.017550
H4K12ac	0.000023	0.000527	0.000920	0.003242	0.000037	0.000656	0.000996	0.003333	0.014945	0.024147
H4K16ac	0.001118	0.003693	0.013658	0.022461	0.004207	0.009103	0.000980	0.003333	0.002210	0.005841
H4K20me1	0.031239	0.044628	0.000019	0.000499	0.003616	0.008157	0.001722	0.004827	0.001307	0.003964
H4K20me2	0.000126	0.001016	0.000002	0.000163	0.000003	0.000163	0.008131	0.015118	0.000048	0.000750
H4K20me3	0.000674	0.002625	0.000217	0.001257	0.000016	0.000465	0.101683	0.126675	0.022932	0.034632
