# Supplementary material for: Proteogenomic characterization and mapping of nucleosomes decoded by Brd and HP1 proteins
Source: Genome Biol. 2012 Aug 16;13(8):R68. doi: 10.1186/gb-2012-13-8-r68 (PMC3491368; doi:10.1186/gb-2012-13-8-r68)
Supplement: Additional file 6 — Table of relative combinatorial PTM abundances determined by quantitative mass spectrometry on the histone H3 peptides (amino acids 9 to 17), (amino acids 18 to 26) and (amino acids 27 to 40) averaged from three independent ChIP experiments with each Brd and HP1 protein and data from three experiments with HEK293 genomic chromatin. [file gb-2012-13-8-r68-S6.PDF]

H3 Peptide	Brd2	Brd3	Brd4	HP1α	HP1β	Genomic
KSTGGKAPR (9-17)						
H3K9unK14un	13.32% ± 2.19%	13.90% ± 0.86%	11.67% ± 0.60%	4.40% ± 1.18%	1.66% ± 1.22%	10.93% ± 1.84%
H3K9me1K14un	12.73% ± 1.95%	11.18% ± 1.12%	9.42% ± 0.66%	6.11% ± 0.58%	5.30% ± 0.38%	10.22% ± 2.14%
H3K9me2K14un	9.39% ± 1.47%	15.25% ± 0.39%	15.91% ± 1.97%	26.31% ± 1.87%	27.36% ± 3.05%	21.60% ± 2.15%
H3K9me3K14un	3.67% ± 0.54%	6.77% ± 0.58%	5.77% ± 0.11%	21.93% ± 0.42%	28.75% ± 1.94%	12.10% ± 1.07%
H3K9acK14un	2.68% ± 0.46%	1.33% ± 0.53%	0.89% ± 0.15%	0.07% ± 0.02%	0.35% ± 0.16%	0.44% ± 0.09%
H3K9unK14ac	16.78% ± 1.72%	12.94% ± 1.00%	13.34% ± 0.29%	2.77% ± 0.74%	1.48% ± 0.13%	12.51% ± 1.23%
H3K9me1K14ac	15.08% ± 0.74%	12.63% ± 1.29%	13.85% ± 1.18%	3.51% ± 0.40%	2.44% ± 0.65%	11.63% ± 2.28%
H3K9me2K14ac	15.74% ± 0.31%	15.20% ± 2.59%	19.22% ± 0.51%	20.78% ± 1.48%	16.10% ± 2.07%	9.48% ± 0.37%
H3K9me3K14ac	4.85% ± 0.51%	6.20% ± 0.83%	6.35% ± 0.48%	13.70% ± 0.43%	16.32% ± 1.74%	9.82% ± 0.56%
H3K9acK14ac	5.76% ± 0.90%	4.61% ± 0.18%	3.57% ± 1.08%	0.42% ± 0.14%	0.24% ± 0.13%	1.25% ± 0.43%
KQLATKAAR (18-26)						
H3K18unK23un	38.92% ± 1.20%	41.94% ± 1.44%	34.88% ± 1.05%	65.99% ± 0.85%	64.80% ± 1.50%	61.17% ± 3.76%
H3K18me1K23un	5.23% ± 1.78%	4.17% ± 0.61%	3.10% ± 0.67%	0.25% ± 0.09%	0.31% ± 0.06%	0.29% ± 0.05%
H3K18unK23me1	0.61% ± 0.34%	0.41% ± 0.20%	0.28% ± 0.15%	5.13% ± 1.65%	4.37% ± 0.43%	0.20% ± 0.03%
H3K18acK23un	5.12% ± 1.36%	3.66% ± 0.19%	7.88% ± 1.17%	2.66% ± 0.29%	2.69% ± 0.66%	1.13% ± 0.19%
H3K18unK23ac	40.88% ± 3.21%	44.10% ± 0.49%	46.23% ± 2.48%	25.40% ± 1.87%	27.31% ± 0.87%	36.09% ± 3.62%
H3K18acK23ac	9.23% ± 0.85%	5.72% ± 0.86%	7.63% ± 0.39%	0.56% ± 0.15%	0.52% ± 0.18%	1.13% ± 0.03%
KSAPATGGVKKPHR (27-40)						
H3K27unK36un	6.95% ± 0.44%	3.61% ± 0.39%	1.47% ± 0.87%	0.85% ± 0.17%	1.63% ± 0.57%	8.10% ± 1.01%
H3K27me1K36un	14.59% ± 0.12%	4.24% ± 1.68%	2.79% ± 0.37%	1.97% ± 0.72%	2.31% ± 0.63%	10.96% ± 0.85%
H3K27me2K36un	18.59% ± 1.23%	13.17% ± 0.32%	17.23% ± 1.48%	20.83% ± 2.09%	28.05% ± 2.72%	15.24% ± 0.74%
H3K27me3K36un	6.48% ± 0.08%	8.87% ± 1.57%	13.09% ± 1.20%	16.64% ± 1.08%	19.27% ± 2.85%	8.42% ± 0.50%
H3K27unK36me1	4.25% ± 0.94%	2.07% ± 0.31%	1.54% ± 0.31%	0.51% ± 0.24%	0.36% ± 0.09%	3.87% ± 0.74%
H3K27unK36me2	3.25% ± 2.47%	2.78% ± 0.73%	1.46% ± 0.09%	1.31% ± 0.21%	1.51% ± 0.42%	4.38% ± 0.85%
H3K27acK36un	0.04% ± 0.01%	0.01% ± 0.01%	0.04% ± 0.02%	0.01% ± 0.01%	0.01% ± 0.00%	0.06% ± 0.01%
H3K27me1K36me1	13.83% ± 2.47%	6.47% ± 1.20%	8.12% ± 2.23%	5.56% ± 1.07%	3.81% ± 0.76%	7.00% ± 1.66%
H3K27me1K36me2	10.38% ± 1.26%	13.56% ± 2.91%	5.28% ± 0.48%	3.46% ± 0.53%	5.79% ± 1.53%	14.73% ± 1.43%
H3K27me1K36me3	2.02% ± 0.47%	3.54% ± 2.85%	7.07% ± 1.89%	2.45% ± 0.78%	2.21% ± 0.31%	3.30% ± 0.62%
H3K27me2K36me1	14.26% ± 1.57%	22.50% ± 3.95%	23.04% ± 2.68%	24.42% ± 4.87%	25.15% ± 1.75%	14.70% ± 1.15%
H3K27me2K36me2	0.98% ± 0.28%	11.36% ± 1.64%	15.68% ± 1.61%	9.72% ± 1.60%	3.13% ± 0.51%	4.94% ± 1.15%
H3K27me3K36me1	2.94% ± 0.56%	5.77% ± 1.25%	1.81% ± 0.27%	11.20% ± 1.45%	6.02% ± 0.53%	3.43% ± 0.23%
H3K27me3K36me2	1.45% ± 0.61%	2.05% ± 0.37%	1.38% ± 0.45%	1.08% ± 0.18%	0.75% ± 0.20%	0.89% ± 0.29%
