# Supplementary material for: Proteogenomic characterization and mapping of nucleosomes decoded by Brd and HP1 proteins
Source: Genome Biol. 2012 Aug 16;13(8):R68. doi: 10.1186/gb-2012-13-8-r68 (PMC3491368; doi:10.1186/gb-2012-13-8-r68)
Supplement: Additional file 7 — P-values from t-tests performed on the fold changes (ChIP/Genomic) from the histone H3 data presented in Additional file 6. t-Tests were performed with data from three independent ChIP experiments for each Brd and HP1 protein and data from three experiments with HEK293 genomic chromatin. P-values were adjusted using the Benjamini-Hochberg correction method to control the false discovery rate (FDR). [file gb-2012-13-8-r68-S7.PDF]

Modification	Brd2		Brd3		Brd4		HP1α		HP1β	
KSTGGKAPR (9-17)	p-value	Adjusted	p-value	Adjusted	p-value	Adjusted	p-value	Adjusted	p-value	Adjusted
H3K9unK14un	0.22166	0.25872	0.064915	0.085172	0.544652	0.587525	0.006635	0.012923	0.0019	0.005131
H3K9me1K14un	0.207576	0.247752	0.528762	0.572053	0.568046	0.603957	0.032414	0.045787	0.017099	0.026681
H3K9me2K14un	0.001258	0.003877	0.007346	0.013895	0.027759	0.040437	0.045818	0.063021	0.055912	0.075227
H3K9me3K14un	0.000261	0.00136	0.001603	0.004634	0.00052	0.002185	0.00012	0.00099	0.000201	0.00124
H3K9acK14un	0.001212	0.003845	0.045523	0.062849	0.011482	0.019691	0.002181	0.005805	0.431809	0.476924
H3K9unK14ac	0.024939	0.037247	0.664826	0.683294	0.316545	0.35817	0.000298	0.001449	0.000101	0.000956
H3K9me1K14ac	0.067644	0.087511	0.546414	0.587713	0.208826	0.248443	0.003714	0.008279	0.002564	0.006241
H3K9me2K14ac	2.28E-05	0.000527	0.019347	0.029581	1.14E-05	0.000352	0.000211	0.001257	0.00552	0.011161
H3K9me3K14ac	0.000347	0.001584	0.003307	0.007697	0.001271	0.003887	0.000695	0.00268	0.003562	0.008085
H3K9acK14ac	0.001437	0.004254	0.000233	0.001301	0.026004	0.038181	0.03233	0.045787	0.017162	0.026681
KQLATKAAR (18-26)										
H3K18unK23un	0.000616	0.002477	0.001164	0.00376	0.000308	0.001469	0.096346	0.120841	0.195377	0.233947
H3K18me1K23un	0.008558	0.015733	0.000379	0.001689	0.001874	0.005097	0.599058	0.631485	0.643819	0.667264
H3K18unK23me1	0.105124	0.129652	0.153596	0.185721	0.413395	0.459328	0.006671	0.012923	7.6E-05	0.000873
H3K18acK23un	0.00736	0.013895	8.62E-05	0.000873	0.000601	0.002446	0.001639	0.004701	0.016817	0.026366
H3K18unK23ac	0.160928	0.193953	0.019122	0.029357	0.016094	0.025557	0.010465	0.018706	0.015035	0.024186
H3K18acK23ac	7.86E-05	0.000873	0.000751	0.002778	9.04E-06	0.000304	0.003004	0.007035	0.003969	0.008689
KSAPATGGVKKPHR (27-40)										
H3K27unK36un	0.144083	0.175943	0.00202	0.005416	0.001	0.003333	0.000258	0.00136	0.000648	0.002552
H3K27me1K36un	0.001834	0.005063	0.003474	0.007934	0.000107	0.000966	0.000153	0.001088	0.000143	0.001081
H3K27me2K36un	0.015701	0.02504	0.011478	0.019691	0.105649	0.129868	0.011945	0.020274	0.001402	0.004182
H3K27me3K36un	0.002622	0.006341	0.660503	0.682643	0.003385	0.007779	0.000282	0.001444	0.002903	0.006841
H3K27unK36me1	0.609137	0.636848	0.017681	0.027259	0.007257	0.01384	0.00169	0.004811	0.001224	0.003845
H3K27unK36me2	0.495066	0.541936	0.069767	0.089944	0.004197	0.009103	0.003793	0.008353	0.006467	0.012728
H3K27acK36un	0.084275	0.107523	0.005133	0.010505	0.298984	0.340382	0.002835	0.006724	0.002369	0.006006
H3K27me1K36me1	0.016509	0.026104	0.677346	0.692315	0.522666	0.568783	0.276987	0.317292	0.038986	0.054229
H3K27me1K36me2	0.016639	0.026198	0.567319	0.603957	0.000404	0.001759	0.000212	0.001257	0.001779	0.004949
H3K27me1K36me3	0.048095	0.065665	0.89323	0.898085	0.030422	0.043647	0.214002	0.252168	0.054531	0.073637
H3K27me2K36me1	0.715248	0.729041	0.030435	0.043647	0.007745	0.014546	0.028314	0.041083	0.000983	0.003333
H3K27me2K36me2	0.004384	0.00943	0.005139	0.010505	0.000708	0.002694	0.013621	0.022461	0.067402	0.087505
H3K27me3K36me1	0.234888	0.27244	0.033786	0.047532	0.001451	0.004261	0.000797	0.002921	0.001484	0.004325
H3K27me3K36me2	0.22638	0.263398	0.01285	0.0215	0.193463	0.232407	0.40242	0.449835	0.526634	0.571421
