# Supplementary material for: Proteogenomic characterization and mapping of nucleosomes decoded by Brd and HP1 proteins
Source: Genome Biol. 2012 Aug 16;13(8):R68. doi: 10.1186/gb-2012-13-8-r68 (PMC3491368; doi:10.1186/gb-2012-13-8-r68)
Supplement: Additional file 12 — Products from PCR reactions were run on 2% agarose gels stained with ethidium bromide and visualized on a Gel Doc XR system (BioRad® Hercules, CA, USA). One half of each PCR reaction was loaded. Gel is labeled corresponding to the templates used for the PCR reactions: control ChIP (beads alone), Brd4 ChIP and ChIP input DNA. [file gb-2012-13-8-r68-S12.PDF]

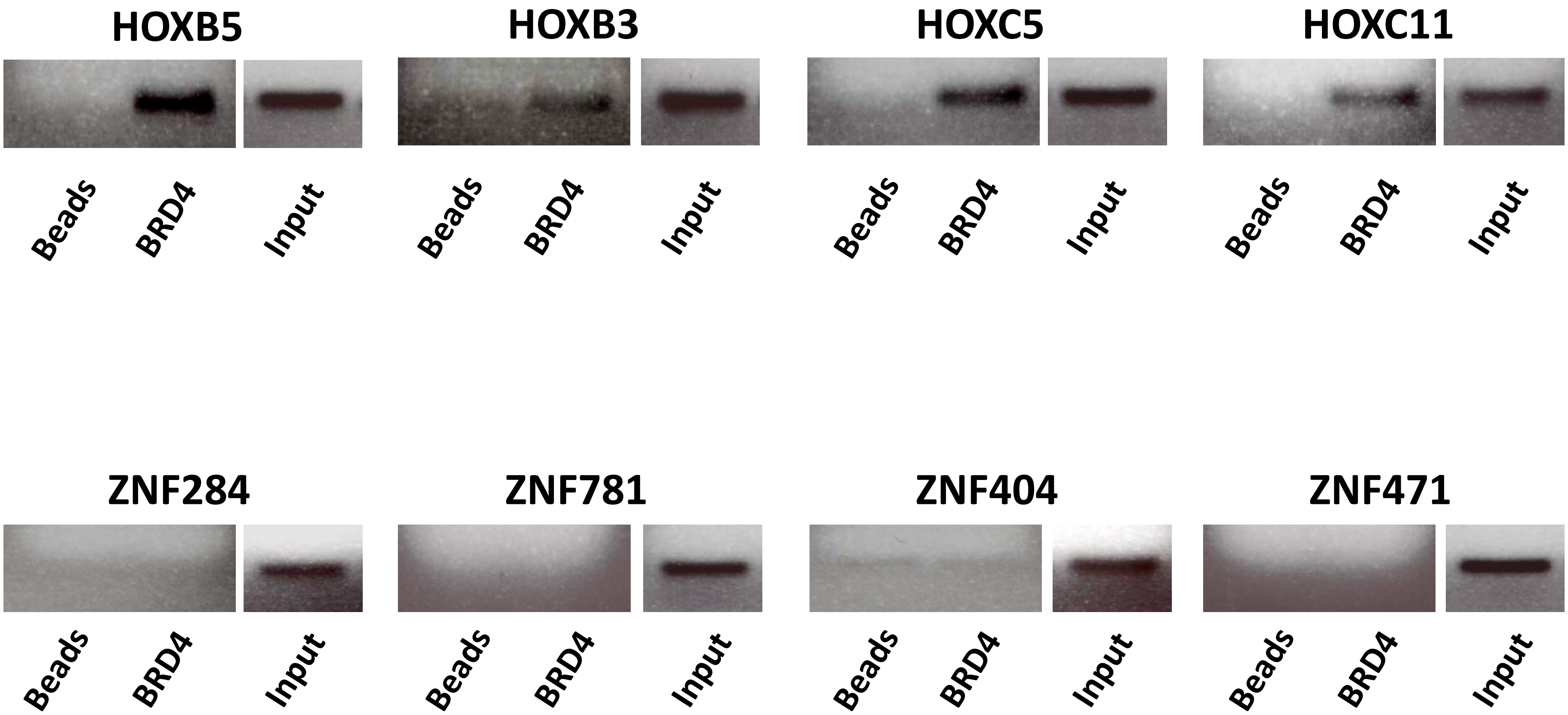
HOXB5
HOXB3
HOXC5
HOXC11
Beads
BRD4
Input
ZNF284
ZNF781
ZNF404
ZNF471
