# Supplementary material for: Proteogenomic characterization and mapping of nucleosomes decoded by Brd and HP1 proteins
Source: Genome Biol. 2012 Aug 16;13(8):R68. doi: 10.1186/gb-2012-13-8-r68 (PMC3491368; doi:10.1186/gb-2012-13-8-r68)
Supplement: Additional file 14 — Supplemental Materials and methods. [file gb-2012-13-8-r68-S14.PDF]

Supplemental Materials and Methods

Native endogenous Brd4 Chromatin Immunoprecipitaion (ChIP).

For the Native ChIP, twenty 10cm plates of 293 cells were harvested at 80-90% confluency (grown under standard growth conditions). Nuclei were harvested by gentle douncing and swelling for 20 min. in 200mls TMSD, hypotonic lysis buffer (40mM Tris pH 7.5, 5mM $MgCl_2$, 20% Sucrose, 1mM DTT, 0.1mM PMSF and 8mM Sodium Butyrate) at 4°C. Nuclei were then harvested by centrifugation in a tabletop centrifuge at 3600 RPM for 10 min. The nuclei were then resuspended in 1 ml of MNase digestion buffer (10mM Hepes pH 7.5, 50mM NaCl, 5mM $MgCl_2$, 5mM $CaCl_2$, 0.1mM PMSF and 8mM Sodium Butyrate). Two Units of MNase were then added and the chromatin was digested to mononucleosomes at 37°C for 37 min. The digestion was then stopped upon the addition of 20mM EGTA and the nuclei were pelleted at 12,000 RPM n a benchtop centrifuge. The supernatant containing the mononucleosomes was collected and the remaining nuclei were resuspended in 1ml of BC50 (40mM Tris pH 7.5, 5mM $MgCl_2$, 50mM NaCl, 5% glycerol, 0.1mM PMSF and 8mM Sodium Butyrate). Nuclei were pelleted again at 12,000 RPM in a benchtop centrifuge and the supernatant containing the remaining mononucleosomes was collected and pooled with first supernatant. To the pooled supernatants 10ul of protein A-agarose beads (previously blocked with salmon sperm DNA and BSA) with coupled Brd4 antibody (mAb Epitomics, 5716) was added and the binding reaction proceeded for 5 hrs at 4°C with rotation. An identical control reaction was done with protein A beads alone. Beads were then collected by pelleting in a benchtop centrifuge at 1000 RPM for 2 mins. The beads were then washed three times with BC50 with repeated centrifugation. The ChIPs were eluted from the beads by incubation with 200ul elution buffer (40mM Tris pH 7.0, 50mM NaCl and 0.2% SDS) at 42°C for 30 minutes. DNA from the ChIP elutions was purified with a PCR cleanup kit (Qiagen ®).

PCR amplification of HOX and ZNF genes.

All PCR were performed with TAQ polymerase following manufacturer's suggested conditions. Each reaction produced approximately a 75 bp product in the 5' region of each target gene. Annealing for PCRs was performed at 55°C and elongation at 72°C, each for 1 minute. The primers used to amplify the target HOX and ZNF genes are listed below.

HOXB5 (5'-CAGGGATCCCGCTGCCATGC-3' and 5'-GTTGACGCTGAGGTCCATCC-3')
HOXB3 (5'-CCCCCATTTCAGGCCGCCAC-3' and 5'- CCAGGGACTGCAGCGAGCAA-3')
HOXC5 (5'-TCCAGGTACTGCTACGGCGG-3' and 5'-GGAGAGAGTTGGAAGGCGCA-3')
HOXC11 (5'-CAGTTGCACTTACTACATGC-3' and 5'-CTGACGAGAGGGGGCCTGGG-3')

ZNF284 (5'-TGTATCGAGATGTCATGCTG-3' and 5'-TCTCGGTGGGAAAGTTGATG-3')
ZNF781 (5'-ATATGTGGAAAGCCCTTTAG-3' and 5'-GTTTCCCACCAGTGTGAATT-3')
ZNF404 (5'-TGATGTTGGAGAATTATACT-3' and 5'-GATAACTTGTTGCTTTCAGT-3')
ZNF471 (5'-CTGCTCAGAAGCGTTTATAC-3' and 5'-CTGCTCAGAAGCGTTTATAC-3')

Western Blots

Western blots were performed following standard protocols. Briefly, proteins were separated on a 10% SDS-PAGE and transferred to Immobilon P membrane (Millipore ®). The membranes were blocked with 10% nonfat dry milk dissolved in standard TBST buffer. Blots were probed with anti-Brd4 (mAb Epitomics, 5716), anti-HP1β (pAb Cell Signaling Tech. 2613) and β-actin control (mAb Santa Cruz, sc-81178) in TBST following manufacturers recommended conditions for each antibody. Blots were then washed 3x with TBST and probed with an appropriate HRP-coupled secondary antibody in TBST for 30 minutes. Blots were again washed 3x with TBST and developed with ECL reagent (Invitrogen ®) and visuallized by chemilluminescence and X-Ray film.

Multiple Hypothesis Testing Corrections

To address issues concerning multiple hypothesis testing, all histone modification abundance t-test p-values (n = 340) were adjusted using the Benjamini-Hochberg method** with an FDR cutoff of 0.01. Both pre-adjustment p-values and post-adjustment p-values (ie. q-values) are presented.
